# The Association of Total Meat Intake with Cardio-Metabolic Disease Risk Factors and Measures of Sub-Clinical Atherosclerosis in an Urbanising Community of Southern India: A Cross-Sectional Analysis for the APCAPS Cohort

**DOI:** 10.3390/nu16050746

**Published:** 2024-03-05

**Authors:** Hemant Mahajan, Poppy Alice Carson Mallinson, Judith Lieber, Santhi Bhogadi, Santosh Kumar Banjara, Vadde Sudhakar Reddy, Geereddy Bhanuprakash Reddy, Bharati Kulkarni, Sanjay Kinra

**Affiliations:** 1 Indian Council of Medical Research—National Institute of Nutrition, Hyderabad 500007, India; santhi_bhogadi@isb.edu (S.B.); santosh.kumar@icmr.gov.in (S.K.B.); sudhakarr.v@icmr.gov.in (V.S.R.); reddyg.bp@icmr.gov.in (G.B.R.); 2 Faculty of Epidemiology and Population Health, London School of Hygiene & Tropical Medicine, London WC1E 7HT, UK; judith.lieber1@lshtm.ac.uk (J.L.); sanjay.kinra@lshtm.ac.uk (S.K.); 3 Indian Council of Medical Research, Delhi 110029, India; kulkarni.bharati@icmr.gov.in

**Keywords:** cardiovascular diseases, diabetes, India, meat, risk factors

## Abstract

Aim: Meat is commonly consumed in India; however, in comparison to Western settings, it is eaten in relatively lower quantities and with minimal processing. The association between meat intake and cardio-metabolic diseases (CMDs) and their risk factors in India is currently uncertain. We examined whether meat intake is associated with risk factors for CMDs and the measures of subclinical atherosclerosis in urbanising villages in southern India. Methods: We conducted a cross-sectional analysis of 6012 adults (52.3% male) participating in the Andhra Pradesh Children and Parents’ Study (APCAPS), which is a large prospective, intergenerational cohort study in Southern India that began with the long-term follow-up of the Hyderabad Nutrition Trial (1987–1990). We used cross-sectional data from the third wave of data collection conducted in 2010–2012, where total meat intake was assessed using 100-item, semi-quantitative validated food frequency questionnaires (FFQ). The FFQs were validated using multiple weighed 24 h dietary recalls. The main predictor, ‘total meat intake’, was calculated as the sum of chicken, red meat, and fish consumption. The risk factors for CMDs [systolic blood pressure (SBP), diastolic blood pressure (DBP), body mass index (BMI), waist circumference (WC), fasting glucose, total cholesterol, homeostasis model assessment insulin resistance (HOMA-IR), total cholesterol, low-density lipoprotein cholesterol (LDL-C), high-density lipoprotein cholesterol, triglycerides, and C-reactive protein] and measures of subclinical atherosclerosis [Carotid Intima-Media Thickness, Pulse Wave Velocity, and Augmentation Index] were assessed using standardised clinical procedures. Stratified by gender, the association of meat intake with the risk factors of CMDs and measures of subclinical atherosclerosis was examined using linear multilevel models with random intercept at the household level. Results: The mean (SD) age of the male (*n* = 3128) and female participants (*n* = 2828) was 34.09 years (15.55) and 34.27 years (12.73), respectively. The median (IQR) intake of meat was 17.79 g/day (8.90, 30.26) in males and 8.90 g/day (4.15, 18.82) in females. In males, a 10 g increase in total meat intake/1000 Kcal/day was positively associated with DBP, BMI, WC, total cholesterol, LDL-C, and triglycerides, whereas in females, a 10 g increase in total meat intake/1000 Kcal/day was positively associated with SBP, DBP, fasting glucose, HOMA-IR, total cholesterol, LDL-C, and triglycerides. There was no relationship between meat consumption and measures of subclinical atherosclerosis. Conclusions: Meat intake had a linear positive association with CMD risk factors among the relatively younger Indian population who were consuming meat at lower levels compared to their European counterparts.

## 1. Introduction

Cardiovascular diseases (CVDs) are the leading cause of death globally and their burden is rising in economically developing countries worldwide, including India. According to the Global Burden of Disease study age-standardised estimates (2010), nearly a quarter (24.8%) of all deaths in India are attributable to CVD. The age-standardised CVD death rate of 272 per 100,000 population in India is higher than the global average of 235 per 100,000 population [1]. The prevalence of CVD in India (both in urban as well as rural areas) has increased by nearly five times in the last three decades, which might be attributed to the increasing prevalence of CVD-risk factors such as type 2 diabetes mellitus (DM), hypertension, hypercholesterolemia, and obesity [2,3]. Primary prevention remains the main strategy to reduce the burden of CVDs. The identification of modifiable factors affecting CVDs and their risk factors may help to inform intervention development and improve public health policies.

Meat can constitute an important component of a healthy balanced diet, supplying essential amino acids and micronutrients with greater bioavailability compared to plant-based diets [4]. However, studies have established the clear association between processed meat intake and CMDs [5,6,7,8,9]; the evidence is largely from Western countries, where meat undergoes relatively high levels of processing (by the addition of high levels of salt and/or chemical preservatives to improve its taste or to extend its shelf life) and is eaten in relatively large quantities. However the association of unprocessed meat (fresh cuts without addition of any preservatives), which is commonly consumed in India and also in lower quantities (~4.0 kg annual intake per person per year) compared to European counterparts (>50.0 kg annual intake per person per year), with CMDs or their risk factors is uncertain and needs further exploration [10,11].

Data from cross-sectional and prospective studies (mainly conducted in Western countries) suggest that individuals who regularly consume meat products tend to have higher blood pressure, body weight (adiposity), insulin resistance, and total and LDL cholesterol and triglycerides [12,13]. Meat products are thought to affect the serum lipid profile due to the high saturated fat content and absence of fibre, leading to increased adiposity. Sodium and nitrite content in meat products has been largely demonstrated to increase blood pressure and peripheral vascular resistance, and to lower arterial compliance [14]. Nitrates and their by-products have also been experimentally demonstrated to promote endothelial dysfunction and atherosclerosis development [15]. 

International dietary guidelines [16] as well as the Sustainable Development Goals [17] recommend a reduced intake of meat and increased intake of a plant-based diet; however, meat consumption is steadily increasing in India. The results of a large, nationally representative survey, the National Family Health Survey (NFHS), demonstrate that the share of meat eaters increased from NFHS-4 to NFHS-5: 70.0% to 71.8% among women and 81.9% to 83.2% among men [18]. Additionally, in the last six years, the daily consumption of fish, chicken, and meat among men has exponentially increased from 1.8% to 8.0%. Per capita availability of meat in India has also increased from 5.32 kg/annum in 2015 to 6.82 kg/annum in 2022 [19].

Both meat consumption and CMDs are rapidly rising in India, due in part to economic development, rapid and unplanned urbanisation, and limited access and availability of nutritious food (fruits, green leafy vegetables, and pulses) [20,21,22]. To the best of our knowledge, there are few studies focusing specifically on the relationship between total meat intake in the Indian population and risk factors of CMDs, and the association remains unclear [23,24,25,26]. The aim of this study is to assess the relationship between total meat intake and the risk factors of CMDs and the measures of sub-clinical atherosclerosis in an urbanising community in southern India; we hypothesised that total meat intake is positively associated with the prevalence of risk factors of CMDs and the measures of subclinical atherosclerosis. We examined the proposed research question using data from the Andhra Pradesh Children and Parents’ Study (APCAPS), which is set in 29 rural villages and towns ~50 km from the metropolitan city of Hyderabad, India.

## 2. Methods

### 2.1. Study Sample

The APCAPS is a large prospective, intergenerational cohort study in Southern India that began with the long-term follow-up of the Hyderabad Nutrition Trial (1987–1990). The Hyderabad Nutrition Trial (1987–1990) was a community-based non-randomised controlled intervention trial conducted in 29 villages of the Ranga Reddy district in Telangana state, India. It evaluated India’s Integrated Child Development Services (ICDS) scheme, which is a long-standing scheme aimed at improving child growth and development through integrated provision of food supplementation, anaemia control, immunisation, education, and basic healthcare to pregnant and lactating women and children up to 6 years. Using the opportunity afforded by the gradual rollout of this nationwide scheme during the 1980s and 1990s, the National Institute of Nutrition of India conducted the trial to assess the effect of food supplementation in pregnancy on offspring’s birth weight [27,28]. 

A cluster of villages was chosen from two adjacent administrative areas (called ‘blocks’), one with the ICDS scheme in place (intervention arm) and the other awaiting implementation at that time (control arm). As the 100 or so villages in each of the two blocks were spread over an unfeasibly large area for data collection, contiguous villages surrounding the geographic centre of each block were selected to make up the planned sample size of 30,000 total population in each block. 

This resulted in 15 intervention and 14 control villages geographically separated by uninvolved villages. The food supplement (‘upma’, a local food prepared from a corn–soya blend and soya bean oil) was offered daily to women throughout pregnancy and lactation (2.51 MJ of energy and 20–25 g of protein daily) and children below the age of 6 years (1.25 MJ and 8–10 g protein daily). A total of 2964 birth weights were recorded within 48 h of delivery with an infant beam balance, with an accuracy of 20 g. 

The offspring born during the trial have been followed up on three times: first follow-up in 2003–2005 (mean age 16 years; N = 1165), second follow-up in 2009–2010 (mean age of 20 years; N = 1446), and third follow-up in 2010–2012 (mean age of 22 years; N = 1360). The third follow-up was conducted primarily to extend the study to parents and siblings of the trial offspring. In the third FU, 6944 of 10,213 (68%) invited family members attended the clinical examination [27,28]. In the present study, we used cross-sectional data from the third wave of APCAPS data collection. 

### 2.2. Diet Assessment

Diet was assessed by a purposively developed and validated (using multiple weighed 24 h dietary recalls) 100-item semi-quantitative food frequency questionnaire (FFQ) [29]. The trained interviewer administered the questionnaire and assessed the average portion size and frequency of the selected local food items consumed over the past year including meat consumption, green leafy vegetables, fruits, legumes, etc. Portion size was assessed by showing examples of utensils (bowl, ladle, tablespoon, teaspoon, glass) to the participants, who were asked to report portion sizes in relation to these standards. Prior to the study, weighed recipes were collected from residents of the study area who were food preparers and combined with Indian food composition tables (or international sources if unavailable) to develop study-specific nutrient databases [29,30,31,32]. The commonly consumed non-vegetarian food included chicken, fish, goat, lamb, and beef. The main predictor ‘total meat intake’ was calculated as the sum of the chicken, red meat (goat, lamb, or beef), and fish consumption. For this study we defined variable ‘antioxidants’ as the sum of vegetables (including green leafy vegetables), fruits, fruit juices, and legumes.

### 2.3. Clinical Assessment

As published elsewhere [27,28], at clinics established within the villages, a trained interviewer collected data related to tobacco and alcohol consumption using standard questions from India’s Third National Family Health Survey [33]. Socioeconomic position was assessed using a subset of 14 questions (out of 29) of the Standard of Living Index (SLI) and applying the prescribed weights; a higher score indicates a higher socioeconomic position. Physical activity was assessed using a validated questionnaire (activities over the past week), which was used to derive metabolic equivalent tasks (METs; expressed in hours/day) and time spent sedentary (minutes/day); its validation against tri-axial accelerometers in this setting has been published [34]. Physical activity level was calculated as total energy expenditure over a 24 h period divided by his/her basal metabolic rate. 

Using standard protocols [28], weight was measured with a digital weighing scale (SECA, www.seca.com) and standing height with a plastic stadiometer (Leicester measure; Chasmors, London, UK). Waist circumference (WC) was measured using a non-stretch metallic tape at the narrowest point of the abdomen between the ribs and the iliac crest. Anthropometric measurements were taken twice and averaged for analyses; where the difference between readings was more than the acceptable level (5 mm for height, 0.5 kg for weight, and 1 cm for WC), a third reading was taken. Body mass index (BMI) was calculated as (weight in kilograms ÷ (height in metres^2^)).

Systolic (SBP) and diastolic blood pressure (DBP) were measured at the right upper arm in the sitting position, using a validated oscillometric device (Omron M5-I model). Participants were asked to rest for 5 min before three readings were taken, each 1 min apart. The mean of the final two readings was used for analysis. Participants with a previous history of hypertension diagnosis by a clinician and/or SBP ≥ 140 mmHg and/or DBP ≥ 90 mmHg were considered as hypertensive.

Venous blood samples were drawn after a minimum of 8 h fasting and centrifuged immediately. Glucose was assayed on the same day using the oxidase-peroxidase enzymatic (GOD-PAP) method. Total cholesterol, triglycerides, and serum high-density lipoprotein cholesterol (HDL-C) were assayed using the enzymatic colorimetric method. Serum insulin was assayed on an e-411 auto-analyser using an electrochemiluminescence immunoassay. High-sensitivity C-reactive protein (CRP) was measured using a particle-enhanced immunoturbidimetry method. The quality of biochemical assays was assured through internal controls and external assurance arrangements with Randox International Quality Assessment Scheme (lipids) and UK National External Quality Control Assessment Service (insulin). Intra- and inter-assay coefficients of variation were <3% and <5%, respectively, for all assays.

Low-density lipoprotein cholesterol (LDL-C) was estimated using the Friedewald–Fredrickson formula (Total cholesterol—HDL-C—(triglycerides/5)) [35]. Homeostasis model assessment insulin resistance (HOMA-IR) score was estimated as: [Fasting Insulin × Fasting Glucose/22.5 [17]. Participants with a previous history of diabetes diagnosis and/or fasting glucose ≥126 mg/dL were considered as diabetic. Quality of clinical measurements was ensured through rigorous protocols, regular standardisation of equipment, and teams to detect any drifts over time [28]. Reproducibility of measurements was evaluated by repeat measurements on a 5% random subsample; the intraclass correlation coefficients were >0.98 for anthropometric measurements, >0.85 for vascular measurements, and >0.94 for biochemical assays.

### 2.4. Assessment of Subclinical Atherosclerosis Measures

A subsample of participants agreed to attend an additional clinic in Hyderabad for examination of subclinical measures of CVDs: Carotid Intima-Media Thickness (CIMT), Pulse Wave Velocity (PWV), and Augmentation Index (AIx). Intima-media thickness of the right common carotid artery (CIMT) close to the bulb was measured by a trained physician following the recommended guidelines, using a B-mode ultrasound scanner (Ethiroli Tiny-16a, Surabhi Biomedical Instrumentation, Coimbatore, Tamil Nadu, India). A semi-automated software (AtheroEdge™, 3.0) was used to read a 10 mm segment from the near (i.e., the artery wall close to the ultrasound probe) and far walls. Our primary outcome was mean CIMT, which was calculated as the mean CIMT of available measurements [28]. 

PWV and Aix (markers of arterial stiffness) were measured in the supine position using a Vicorder device (Skidmore Medical Limited, Bristol, UK) [28]. Carotid and femoral artery pressure waveforms were recorded simultaneously by placing BP cuffs around the neck (30 mm wide cuff) and upper thigh (100 mm wide cuff) with the subjects in the supine position. The cuffs were inflated to 60 mmHg, and pressure waveforms were recorded for 3 s using a volume displacement method. The foot of the pressure waveform was identified using a cross-correlation algorithm centred at the peak of the second derivative of pressure. The difference in time between pulse arrival at the carotid artery in comparison with the femoral artery was taken as the ‘transit time’. The difference in distance between the two sites was measured using a tape measure from the upper edge of the femoral cuff. PWV was calculated by dividing the ‘difference in distance’ by the ‘transit time’ in meters per second (m/s). PWV was measured three times, and the average was used for all further analysis [36]. AIx was derived from the central pressure waveform measured over the radial artery in the supine position. AIx was defined as the difference between the first and second peaks of the central arterial waveform and expressed as a percentage of central pulse pressure [37]. AIx was measured twice and the average of two high quality recordings (quality index > 90%) was used for all further analysis [38]. 

### 2.5. Statistical Analyses

We assessed data on the following cardiovascular risk factors as outcomes: SBP, DBP, BMI, WC, fasting total cholesterol, HOMA-IR, total cholesterol, LDL-C, HDL-C, triglycerides, CRP, CIMT, PWV, and AIx. All the analyses were stratified by sex as the meat intake was remarkably different between the genders. We restricted all analyses to participants with complete exposure and outcome data. For SBP, DBP, BMI, WC, fasting glucose, HOMA-IR, total cholesterol, LDL-C, HDL-C, triglycerides, and CRP, we analysed data for 3184 males and 2828 females. For CIMT, PWV, and AI, we analysed data for 1730 males and 1573 females, 1707 males and 1570 females, and 1631 males and 1480 females, respectively. Before analysis, we checked distributions of outcome variables and applied a log transformation to the values of skewed variables (fasting glucose, HOMA-IR, triglycerides, CRP, PWV, and AI). After first checking for evidence of non-linearity in its association with CVD risk factors, we used total meat intake as a linear exposure and calculated results as β-coefficients (95% Confidence Interval) per one SD of meat intake, as well as per 10 g of meat intake/1000 Kcal/day.

To evaluate the adjusted association of meat intake with CMD risk factors, we used linear multilevel modes with random intercept at the household level to account for the potential correlation of CVD risk factors among the members of the same household. We used meat consumption as a continuous variable (per standard deviation increase in meat consumption per day). Additionally, we used the nutrient density method to adjust for caloric intake [39], where we divided 10 g of meat intake by a standardised calorie intake (grams per 1000 kcal) in addition to including energy as a continuous covariate in the model because it can be independently associated with CMD risk factors [40]. 

In the multivariable model, we first calculated age-adjusted (Model 1) β-coefficients (95% Confidence Interval) for total meat intake with CMD risk factors. We further adjusted for socio-demographic (Model 2: Model 1 + occupation and SLI) and lifestyle variables (Model 3: Model 2 + antioxidants, physical activity, tobacco and alcohol consumption), and energy intake (Model 4: Model 3 + energy) to assess the independent association of meat intake and CMD risk factors. We repeated the analyses in the following ways: (i) by excluding study participants (87 males and 153 females) who were not consuming any type of meat; (ii) by excluding participants diagnosed with CVD or diabetes mellitus or hypertension. In secondary analyses, we evaluated the individual effect of red meat and chicken on CMD risk factors for all participants. 

To mitigate the risk of type 1 errors (i.e., false positives) due to performing multiple statistical tests, we reported *p* values that are significant at a false discovery rate of 5% using the Benjamini–Hochberg method [41]. For this, we considered all models with the same exposure as part of the same family of tests (i.e., 14 tests per family). Results from secondary analyses were undertaken as exploratory analyses with two-tailed *p*-value < 0.05 as significant.

## 3. Results

We analysed data for 6012 (87%) participants, with complete data for all exposure and outcome variables except CIMT, PWV, and AIx. For CIMT, PWV, and AIx the complete data were available for 3303 (47.5%), 3277 (47.2%), and 3111 (44.8%) participants, respectively. The mean (SD) age of the male participants (*n* = 3128) was 34.09 years (15.55), and of female participants (*n* = 2828) was 34.27 years (12.73). The median (IQR) intake of meat was 17.79 g/day (8.90, 30.26) in males and 8.90 g/day (4.15, 18.82) in females. Males compared to females were more educated and involved in skilled and professional occupations, more frequently daily drinkers (9.6% vs. 2.4%), more frequently current tobacco consumers (32.0% vs. 11.3%), physically less active (1.58 vs. 1.65), and consumed more calories (2443.95 vs. 1810.93 Kcal) and antioxidants (222.99 g vs. 186.57 g). Males compared to females had higher SBP (121.93 vs. 115.18 mmHg), higher hypertension burden (21.1% vs. 13.8%), less insulin resistance (1.11 vs. 1.25), lower HDL-C (42.54 vs. 45.03 mg/dL), lower LDL-C (93.89 vs. 98.74 mg/dL), higher median triglycerides (106.4 vs. 93.7 mg/dL), lower total cholesterol (161.41 vs. 165.36), lower BMI (20.37 vs. 20.83 kg/m^2^), higher WC (736.15 vs. 691.53 mm), lower CRP (0.90 vs. 1.03 mg/dL), lower CIMT (0.788 vs. 0.847 mm), and lower Aix (21.11% vs. 23.99%) (Table 1).

### 3.1. Primary Outcomes: Males

In males, in an age- and socio-economic status-adjusted model (Model 2), total meat intake was directly associated with DBP, BMI, WC, total cholesterol, LDL-C, triglycerides, HOMA-IR score, and AIx, but was not associated with SBP, fasting glucose, HDL-C, CRP, CIMT, and PWV (Table 2). After further adjustment for behavioural risk factors and total calorie intake (Model 4), total meat intake was directly associated with BMI, WC, total cholesterol, LDL-C, triglycerides (log), and AIx (log), with beta coefficients (95% CIs) of 0.31 kg/m^2^ (0.18 to 0.44), 7.58 mm (3.99 to 11.16), 2.35 mg/dL (0.97 to 3.73), 1.57 mg/dL (0.41 to 2.73), 0.030 mg/dL (0.012 to 0.049), and 0.033% (0.006 to 0.060), respectively, per SD (27.72 g/day) increase in total meat intake. A 10 g increase in total meat intake per 1000 Kcal per day (Model 4) was directly associated with DBP, BMI, WC, total cholesterol, LDL-C, and triglycerides (log), with beta coefficients (95% CIs) of 0.71 mmHg (0.18, 1.24), 0.43 kg/m^2^ (0.28 to 0.58), 10.65 mm (6.57 to 14.72), 3.39 mg/dL (1.81 to 4.97), 2.31 mg/dL (0.99 to 3.63), and 0.032 mg/dL (0.012 to 0.053), respectively (Table 2).

The findings were similar when restricting the sample to only those that ate meat. In the fully adjusted model (Model 4), total meat intake was directly associated with BMI, WC, total cholesterol, LDL-C, triglycerides (log), and AI (log), with beta coefficients (95% Cis) of 0.32 kg/m^2^ (0.19 to 0.46), 7.88 mm (4.21 to 11.55), 2.32 mg/dL (0.91 to 3.72), 1.52 mg/dL (0.34 to 2.70), 0.032 mg/dL (0.012 to 0.050), and 0.029% (0.003 to 0.056), respectively, per SD (27.79 g/day) increase in total meat intake. A 10 g increase in total meat intake per 1000 Kcal per day (Model 4) was directly associated with DBP, BMI, WC, HOMA-IR (log), total cholesterol, LDL-C, and triglycerides (log), with beta coefficients (95% CIs) of 0.75 mmHg (0.20, 1.30), 0.46 kg/m2 (0.31 to 0.61), 11.27 mm (7.10 to 15.44), 0.050 (0.009 to 0.091), 3.35 mg/dL (1.74 to 4.95), 2.26 mg/dL (0.92 to 3.61), and 0.034 mg/dL (0.013 to 0.056), respectively (Supplementary Table S1). 

The findings were similar when analyses were repeated for participants without a history of CVD or DM or hypertension (Supplementary Table S3).

### 3.2. Secondary Outcome: Males

Among males, each additional intake of 22.13 g of chicken per day was directly associated with BMI, WC, total cholesterol, and LDL cholesterol, with beta coefficients (95% CIs) of 0.22 kg/m^2^ (0.09 to 0.36), 4.69 mm (1.03 to 8.35), 2.31 mg/dL (0.90 to 3.73), and 1.59 mg/dL (0.41 to 2.78), respectively, after adjustment for socio-demographic risk factors, behavioural risk factors, total calorie intake, and red meat intake. Each additional intake of 9.74 g of red meat per day (Model 4) was directly associated with DBP, BMI, WC, and triglycerides (log), with beta coefficients (95% CIs) of 0.48 mmHg (0.01 to 0.94), 0.14 kg/m^2^ (0.01 to 0.27), 4.66 mm (1.09 to 8.23), and 0.024 mg/dL (0.006 to 0.043); and indirectly with HDL, with beta coefficients (95% CIs) of 0.48 mg/dL (0.95 to 0.01) (Table 3).

### 3.3. Primary Outcomes: Females

In females, in an age- and socio-economic status-adjusted model (Model 2), total meat intake was directly associated with WC, HOMA-IR score, total cholesterol, and LDL-C, but was not associated with SBP, DBP, fasting glucose, HDL-C, triglycerides, CRP, CIMT, PWV, and AIx. After adjustment for behavioural risk factors and total calorie intake (Model 4), total meat intake was directly associated with HOMA-IR score (log), total cholesterol, and LDL cholesterol, with beta coefficients (95% CIs) of 0.044 (0.010 to 0.077), 2.22 mg/dL (0.84 to 3.61), 1.67 mg/dL (0.52 to 2.82), respectively, per SD (14.28 g/day) increase in total meat intake. A 10 g increase in total meat consumption per 1000 Kcal per day (Model 4) was directly associated with SBP, DBP, fasting glucose (log), HOMA-IR (log), total cholesterol, LDL-C, and triglycerides (log), with beta coefficients (95% CIs) of 1.04 mmHg (0.33, 1.75), 0.80 mmHg (0.21, 1.39), 0.012 mg/dL (0.003 to 0.021), 0.075 (0.029 to 0.121), 2.97 mg/dL (1.08 to 4.86), 2.36 mg/dL (0.79 to 3.93), and 0.034 mg/dL (0.010 to 0.058), respectively (Table 4).

When we restricted analyses to only meat eaters, total meat intake (Model 4) was directly associated with total cholesterol and LDL-C, with beta coefficients (95% CIs) of 2.21 mg/dL (0.77 to 3.66) and 1.68 mg/dL (0.48 to 2.88), respectively, per SD (14.72 g/day) increase in total meat intake. A 10 g increase in total meat consumption per 1000 Kcal per day (Model 4) was directly associated with SBP, DBP, HOMA-IR score (log), total cholesterol, LDL-C, and triglycerides (log), with beta coefficients (95% CIs) of 0.98 mmHg (0.24, 1.72), 0.71 mmHg (0.10, 1.33), 0.073 (0.025, 0.120), 3.10 mg/dL (1.12 to 5.07), 2.47 mg/dL (0.83 to 4.11), and 0.034 (0.008, 0.059) mg/dL, respectively (Supplementary Table S2). 

The findings were similar when analyses were repeated for participants without a history of CVD or DM or hypertension (Supplementary Table S4).

### 3.4. Secondary Outcome: Females

Among females, a 1 SD (10.86 g/day) increase in chicken intake was positively associated with HOMA-IR (log), total cholesterol, LDL cholesterol, triglycerides (log), and AI (log), with beta coefficients (95% CIs) of 0.057 (0.024, 0.090), 2.15 mg/dL (0.80 to 3.51), and 1.66 mg/dL (0.54 to 2.78), 0.020 mg/dL (0.002 to 0.037), and 0.026% (0.001 to 0.052), respectively, after adjustment for behavioural risk factors, total calorie intake, and red meat intake (Model 4). The red meat intake, however, showed no evidence of association with any of the CVD risk factors (Table 5).

## 4. Discussion

In this study, we found that among males, a 10 g increase in total meat consumption per 1000 Kcal per day was positively associated with DBP, BMI, WC, total cholesterol, LDL cholesterol, and triglycerides, whereas among females, a 10 g increase in total meat consumption per 1000 Kcal per day was directly associated with SBP, DBP, fasting glucose, HOMA-IR, total cholesterol, LDL-C, and triglycerides. However, there was no association between total meat intake and measures of subclinical atherosclerosis in males (except AIx) as well as females. The findings were very similar when we analysed data only for meat eaters, as well as excluding participants diagnosed with CVD, DM, or hypertension. These findings may support the idea that meat consumption, even at lower levels, among Indians could increase the levels of CMD risk factors. Moreover, the linear association suggests that even a small reduction in meat intake would lead to a reduction in CMD risk factors. This is a novel finding, as this is the first study that has assessed the association of meat intake with risk factors of CMDs and measures of subclinical atherosclerosis at a community level in an Indian setting.

### 4.1. Comparison with Previous Studies

Epidemiological studies, conducted mainly in high-income countries, have shown the positive association of red and processed meat intake with increased risk of CMDs, some cancers, and mortality [5,6,7,8,9]. The association appears to be non-linear, and absent below 0.5 servings/day (~50 g), prompting dietary guidelines in many high-income countries to recommend the intake of red and processed meat to be less than 50–70 g daily [42,43]. Similar guidelines for India do not exist, but limited epidemiological data suggests that the threshold for adverse cardio-metabolic effects of meat intake may be lower for Indians [22]. Our results support this notion and are similar to previous studies conducted in India. In the Indian Migration Study (2005–2007)—a cross-sectional study of 7067 adults (mean age 51 years) from four regions of India—animal-food dietary pattern as well as its individual components (e.g., fish, red meat, and poultry) were associated with increased blood pressure, fasting blood lipids, and glucose, despite a median meat intake of 20 g (inter-quartile range: 10 g to 39 g) per day [26]. In the cross-sectional analyses of data on 156,317 adults (aged 20–49 years) who took part in India’s third National Family Health Survey (2005-06), weekly intake of meat or fish was associated with increased prevalence of diabetes [24,25]. Several studies have also highlighted the lower risk of CMDs amongst vegetarians in India, despite relatively small quantities of meat consumed by non-vegetarians in the same studies [23].

### 4.2. Possible Mechanisms or Pathways

Several pathways have been proposed for increased risk of CMDs associated with meat intake (generally for red meat, although none are specific to red meat alone) and these have varying degrees of relevance to India [44]. 

Meat intake (irrespective of type or dietary pattern) increases the risk of CVD by increasing type 2 diabetes mellitus. Meat intake increases insulin resistance (a precursor of diabetes mellitus) [45] due to (i) higher content of specific amino acids (such as branched chain amino acids) and fat; (ii) more deposition of visceral fat resulting in increased proinflammatory cytokines [46]; (iii) more fat accumulation within muscle and liver cells [47]; (iv) greater presence of haem iron, a pro-oxidant that encourages the production of reactive oxygen species and may damage insulin-producing pancreatic cells [48]; (v) presence of Nitrates and their by-products such as peroxy-nitrite [49]. Among several CVD risk factors, such as blood cholesterol, blood pressure, etc., fasting plasma glucose/insulin resistance has been reported to be linearly and significantly associated with the risk of CVD at all concentrations, which might represent a more effective preventive strategy for cardiovascular risk assessment and prevention than focusing on specific cut-offs. Risk factors related to diet such as meat intake are particularly readily modifiable, unlike other risk factors such as family history, race, and even body weight, etc. Population-level reduction in meat consumption could have a substantial impact on dysglycemia and insulin resistance and subsequently on incidence of CVDs. In this study, total meat consumption is linearly and positively associated with fasting glucose as well as insulin resistance in females but not males. This discrepancy needs further investigations.

Other pathways for increased risk of CMDs associated with meat intake include the following: Processed meats are high in sodium and pro- inflammatory nitrosamines; however, less than 1% of meat consumed in India is processed. Certain cooking practices involving dry heat (e.g., barbecuing and roasting) generate inflammation-inducing advanced glycation products, but these are not more common in India than elsewhere. On the other hand, excessive use of saturated fats in the cooking and frying of food (which can generate trans-fats) is common [25]. Meat quality (e.g., fat, lean, and connective tissue content) is known to vary by nutritional status of the animal and rearing practices, including the use of antibiotics [50].

Several studies have suggested a key role for gut microbiota [51,52]. Meats are high in nutrients such as phosphatidylcholine, choline, and l-carnitine, which are metabolised with the help of gut microbiota to eventually produce trimethylamine-N-oxide (TMAO), which is pro-inflammatory and pro-atherogenic. Red meat consumption is associated with colonisation by gut microbiota that favour greater TMAO production. More recently, red meat intake in mice was shown to promote colonisation by gut microbiota that promote the release of N-glycolylneuraminic acid (Neu5Gc), which when incorporated into endogenous glycoconjugates act as xeno-autoantigens to induce inflammation, but its relevance to humans needs further investigation [52]. Meat eaters have greater iron stores because haem iron (available only in meat) is more easily absorbed and the bioavailability of non-haem iron from animal foods is also higher [53]. The pro-oxidant state induced by the high iron status of meat eaters may increase the risk of CMDs by the formation of reactive oxygen species and by exacerbating the effects of other pathways which are mainly mediated through inflammation [48].

### 4.3. Lowered Threshold for CVDs in Indians

The CVD epidemic in India has some special characteristics compared to European counterparts, such as an earlier age of onset, higher case fatality, and premature mortality [1]. Data show that in addition to traditional risk factors, socio-demographic features, foetal programming, and early life influences contribute to the CVD epidemic. These foetal programming and early life influences may result in differences in genetic susceptibility, glucose and lipid metabolism, and inflammatory states, lowering the thresholds at which conventional risk factors, including meat, for CVD operate [22,54,55,56].

### 4.4. Strengths and Limitations

Our study has several strengths. First, the large sample size was sufficient for evaluating the association of total meat intake with multiple CVD risk factors and measures of subclinical atherosclerosis, including accounting for multiple potential confounders, and stratified by gender. Second, assessment of meat intake was carried out using a validated FFQ coupled with locally collected recipes, specifically designed to capture typical food consumption in the study setting. Third, we assessed outcomes (CVD risk factors and measures of subclinical atherosclerosis) using validated techniques. The findings of this study, however, should be interpreted in the light of several limitations. First, the possibility of response bias in diet data collection and therefore the misclassification of the consumption of meat cannot be completely eliminated. The participants might have hidden their meat consumption to maintain socially acceptable behaviour and avoid any social stigma, as meat consumption is not very common in this area. However, the interviewer was highly trained to conduct interviews, and the interviews were conducted maintaining the privacy of the participants. If anything, the misclassification of meat consumption would have increased the number of type two errors and reduced the strength of association. 

However, the FFQs we used were validated as capturing the local dietary pattern and were administered by a trained interviewer. If present, we expect any exposure misclassification would mostly have been ‘non-differential’, thereby underestimating the strength of the association of meat consumption with CVD risk factors and subclinical atherosclerotic measures. Second, we cannot establish the temporality (i.e., direction of causation) of the association between meat intake and risk factors of CVD and measures of subclinical atherosclerotic based on a cross-sectional analysis. Future longitudinal studies are warranted to explore the role of the consumption of meat and the onset and progression CMDs. Third, although we have controlled for a variety of socio-demographic and clinical characteristics, the possibility of residual confounding cannot be excluded. Fourth, as we have examined the research question in a relatively young and healthy population living in rural and urbanising villages of India, caution may be needed when generalising these results to other populations. 

### 4.5. Public Health Implications

Meat intake, while still low by global standards, is rising steadily in India. Understanding the health effects of meat intake is of immense relevance to health, agriculture, and environmental policies in India. Policy makers are struggling to identify optimal strategies for managing the nutrition transition in India. Meat is a dense source of many important nutrients and could be an important part of the strategy to combat malnutrition and improve the cognitive development of children. On the other hand, CMDs are now the leading cause of death and disability in India, and generally occur at younger ages, thereby accounting for considerable health expenditure and loss of economic productivity. The social and economic impact of CMDs disproportionately affects the poor, women, and the rural populations, for whom the loss of working days and out-of-pocket expenditures associated with these conditions (in the absence of adequate healthcare) can often push them into catastrophic poverty. Our data show a linear positive association of total meat intake with CVD risk factors among a relatively young population who were consuming meat at lower levels compared to their European counterparts. Further research involving long-term follow-up on the participants in studies in which meat intake is assessed over a period of time to see its (meat intake) association with CVD is required to inform policies surrounding meat intake.

## 5. Conclusions

In this community-based, large cross-sectional study, among male participants, the consumption of meat intake had a linear positive association with blood pressure, BMI, WC, total cholesterol, LDL-C, and triglycerides. Among females, the consumption of meat intake had a linear positive association with fasting glucose, insulin resistance, total cholesterol, LDL-C, and triglycerides. Our findings, coupled with evidence from other studies to date, suggest that policy makers should consider the detrimental effect of meat intake on risk factors of CVDs (while formulating the nutritional guidelines surrounding meat intake) even at low levels in the case of Indians. 

## Figures and Tables

**Table 1:** Distribution of socio-demographics, diet variables, cardiometabolic disease risk factors, and measures of subclinical atherosclerosis (*n* = 6012).

Characteristics		Overall (*n* = 6012)	Male (*n* = 3184)	Female (2828)
Socio-demographic				
Age, year (mean, SD)		34.17 (14.29)	34.09 (15.55)	34.27 (12.73)
Education (*n*, %)	No formal education	2120 (35.3)	749 (23.5)	1371 (48.5)
	Primary	1417 (23.6)	817 (25.7)	600 (21.2)
	Secondary	2009 (33.4)	1294 (40.6)	715 (25.3)
	Beyond secondary	466 (7.7)	324 (10.2)	142 (5.0)
Occupation (*n*, %)	Unemployed and unskilled labourer	2533 (42.1)	1186 (37.2)	1347 (47.6)
	Housewife and retired and student	1889 (31.4)	738 (23.2)	1151 (40.7)
	Manual (semi-skilled and skilled)	1265 (21.0)	998 (31.3)	267 (9.4)
	Skilled non-manual and Semi-professional and professional	325 (5.4)	262 (8.2)	63 (2.2)
Standard of Living Index (mean, SD)		28.65 (8.43)	29.10 (8.34)	28.14 (8.50)
Dietary and Behavioural				
Total meat intake, g/day (mean, SD)		19.59 (23.05)	24.62 (27.72)	13.92 (14.28)
Total meat intake, g/day (median, IQR)		14.56 (6.11, 25.31)	17.79 (8.90, 30.26)	8.90 (4.15, 18.82)
Chicken intake, g/day (median, IQR)		10.40 (3.94, 19.30)	15.91 (7.53, 23.38)	8.43 (3.15, 16.87)
Red meat intake, g/day (median (IQR)		1.42 (0.43, 5.28)	2.64 (0.66, 6.90)	0.92 (0.22, 3.41)
Calorie intake, kcal/day (median, IQR)		2102.38 (1626.33, 2713.28)	2443.95 (1903.59, 3111.90)	1810.93 (1447.83, 2224.59)
Antioxidants, g/day (median, IQR)		207.14 (135.98, 324.44)	222.99 (148.46, 347.94)	186.57 (124.28, 295.17)
Current tobacco consumption (*n*, %)		1338 (22.3)	1019 (32.0)	319 (11.3)
Alcohol (*n*, %)	Never	2558 (42.6)	1663 (52.2)	895 (31.7)
	Sometimes	3079 (51.2)	1215 (38.2)	1864 (65.9)
	Daily	375 (6.2)	306 (9.6)	69 (2.4)
Physical activity level (mean, SD)		1.61 (0.21)	1.58 (0.21)	1.65 (0.21)
Moderate to vigorously active (*n*, %)		1816 (30.2)	801 (25.2)	1015 (35.9)
CVD Risk Factors				
SBP, mmHg (mean, SD)		118.75 (15.70)	121.93 (16.07)	115.18 (14.46)
DBP, mmHg (mean, SD)		77.53 (12.67)	79.30 (13.20)	75.53 (11.74)
Hypertension (*n*, %)		1063 (17.9)	672 (21.1)	391 (13.8)
Fasting plasma glucose, mg/dL (mean, SD)		93.51 (20.71)	94.10 (20.78)	92.85 (20.61)
Diabetes (*n*, %)		229 (3.8)	126 (4.0)	103 (3.6)
HOMA-IR (median, IQR)		1.17 (0.68, 1.92)	1.11 (0.61, 1.86)	1.25 (0.75, 1.96)
HDL-C, mg/dL (mean, SD)		43.72 (12.80)	42.54 (12.93)	45.03 (12.52)
LDL-C, mg/dL (mean, SD)		96.17 (30.97)	93.89 (30.97)	98.74 (30.78)
Triglyceride, mg/dL (median, IQR)		100.70 (74.60, 141.20)	106.40 (78.95, 152.3)	93.7 (70.8, 129.05)
Total cholesterol, mg/dL (mean, SD)		163.27 (37.78)	161.41 (37.97)	165.36 (37.46)
BMI, kg/m^2^ (mean, SD)		20.59 (3.80)	20.37 (3.57)	20.83 (4.03)
WC, mm (mean, SD)		715.16 (103.96)	736.15 (100.67)	691.53 (102.55)
C-reactive protein, mg/dL (median, IQR)		0.96 (0.39, 2.47)	0.90 (0.38, 2.31)	1.03 (0.41, 2.61)
CIMT, mm (mean, SD) (*n* = 3303)		0.816 (0.25)	0.788 (0.25)	0.847 (0.24)
PWV, m/s (mean, SD) (*n* = 3277)		6.871 (1.46)	6.870 (1.55)	6.872 (1.34)
AIx, % (mean SD) (*n* = 3111)		22.48 (11.05)	21.11 (10.65)	23.99 (11.30)

AIx, Augmentation Index; BMI, body mass index; CIMT, Carotid Intima-Media Thickness; DBP, diastolic blood pressure; HDL-C, high-density lipoprotein cholesterol; HOMA-IR, homeostasis model assessment insulin resistance; LDL-C, low-density lipoprotein cholesterol; SD, standard deviation; SBP, systolic blood pressure; PWV, Pulse Wave Velocity; WC, waist circumference. Data were analysed for 3184 males and 2828 females for all variables except CIMT, PWV, and AIx. For CIMT, PWV, and AIx, data were analysed for 1730 males and 1573 females, 1707 males and 1570 females, and 1631 males and 1480 females, respectively.

**Table 2:** Association between total meat intake and CMD risk factors and measures of subclinical atherosclerosis among all males: β-coefficients (95% confidence interval) per SD change and 10 g per 1000 kcal per day of meat intake.

CVD Risk Factor	Regression Models	Per SD Change		10 g/1000 kcal/Day	
		β-Coef (95% CI)	*p*-Value	β-Coef (95% CI)	*p*-Value
SBP (*n* = 3184)	Model 1	0.67 (0.15, 1.18)	0.011 *	0.96 (0.32, 1.60)	0.003 *
	Model 2	0.45 (−0.07, 0.96)	0.091	0.70 (0.59, 1.34)	0.032
	Model 3	0.31 (−0.24, 0.85)	0.270	0.51 (−0.15, 1.16)	0.132
	Model 4	0.18 (−0.39, 0.75)	0.546	0.52 (−0.13, 1.17)	0.117
DBP (*n* = 3184)	Model 1	0.99 (0.57, 1.41)	<0.001 *	1.15 (0.63, 1.68)	<0.001 *
	Model 2	0.75 (0.33, 1.18)	0.001 *	0.87 (0.33, 1.41)	0.001 *
	Model 3	0.61 (0.17, 1.06)	0.007 *	0.71 (0.17, 1.25)	0.010 *
	Model 4	0.49 (0.02, 0.97)	0.041	0.71 (0.18, 1.24)	0.009 *
BMI (*n* = 3184)	Model 1	0.55 (0.43, 0.67)	<0.001 *	0.61 (0.46, 0.77)	<0.001 *
	Model 2	0.43 (0.31, 0.55)	<0.001 *	0.47 (0.32, 0.62)	<0.001 *
	Model 3	0.42 (0.29, 0.55)	<0.001 *	0.42 (0.27, 0.57)	<0.001 *
	Model 4	0.31 ^a^ (0.18, 0.44)	<0.001 *	0.43 ^b^ (0.28, 0.58)	<0.001 *
WC (*n* = 3184)	Model 1	14.91 (11.57, 18.25)	<0.001 *	16.56 (12.38, 20.74)	<0.001 *
	Model 2	11.07 (7.78, 14.35)	<0.001 *	11.94 (7.84, 16.04)	<0.001 *
	Model 3	10.57 (7.12, 14.00)	<0.001 *	10.41 (6.31, 14.51)	<0.001 *
	Model 4	7.58 (3.99, 11.16)	<0.001 *	10.65 (6.57, 14.72)	<0.001 *
Fasting Glucose (log) (*n* = 3184)	Model 1	−0.002 (−0.007, 0.004)	0.604	−0.001 (−0.007, 0.007)	0.970
	Model 2	−0.004 (−0.010, 0.002)	0.188	−0.003 (−0.010, 0.004)	0.438
	Model 3	−0.004 (−0.010, 0.003)	0.259	−0.004 (−0.011, 0.004)	0.323
	Model 4	−0.003 (−0.009, 0.003)	0.351	−0.004 (−0.011, 0.004)	0.317
HOMA-IR (log) (*n* = 3184)	Model 1	0.054 (0.022, 0.086)	0.001 *	0.066 (0.026, 0.106)	0.001 *
	Model 2	0.036 (0.004, 0.069)	0.027 *	0.045 (0.004, 0.085)	0.030
	Model 3	0.037 (0.004, 0.071)	0.029	0.038 (−0.002, 0.078)	0.064
	Model 4	0.029 (−0.006, 0.064)	0.104	0.039 (−0.002, 0.079)	0.059
Total cholesterol (*n* = 3184)	Model 1	3.33 (2.08, 4.58)	<0.001 *	4.22 (2.66, 5.78)	<0.001 *
	Model 2	2.90 (1.64, 4.16)	<0.001 *	3.70 (2.13, 5.28)	<0.001 *
	Model 3	2.93 (1.61, 4.26)	<0.001 *	3.33 (1.75, 4.91)	<0.001 *
	Model 4	2.35 (0.97, 3.73)	0.001 *	3.39 (1.81, 4.97)	<0.001 *
HDL-C (*n* = 3184)	Model 1	−0.09 (−0.52, 0.33)	0.669	0.13 (−0.41, 0.66)	0.639
	Model 2	0.09 (−0.34, 0.52)	0.695	0.34 (−0.20, 0.88)	0.217
	Model 3	0.15 (−0.30, 0.60)	0.512	0.41 (−0.13, 0.96)	0.135
	Model 4	0.12 (−0.36, 0.59)	0.635	0.42 (−0.13, 0.96)	0.132
LDL-C (*n* = 3184)	Model 1	2.29 (1.25, 3.33)	<0.001 *	3.12 (1.82, 4.43)	<0.001 *
	Model 2	1.81 (0.76, 2.87)	0.001 *	2.56 (1.25, 3.88)	<0.001 *
	Model 3	1.87 (0.76, 2.97)	0.001 *	2.28 (0.96, 3.60)	0.001 *
	Model 4	1.57 (0.41, 2.73)	0.008 *	2.31 (0.99, 3.63)	0.001 *
TG (log) (*n* = 3184)	Model 1	0.046 (0.029, 0.062)	<0.001 *	0.044 (0.023, 0.064)	<0.001 *
	Model 2	0.041 (0.024, 0.057)	<0.001 *	0.037 (0.017, 0.058)	<0.001 *
	Model 3	0.038 (0.021, 0.055)	<0.001 *	0.032 (0.011, 0.053)	0.003 *
	Model 4	0.030 (0.012, 0.049)	0.001 *	0.032 (0.012, 0.053)	0.002 *
CRP (log) (*n* = 3184)	Model 1	−0.001 (−0.046, 0.043)	0.959	0.042 (−0.014, 0.097)	0.140
	Model 2	−0.015 (−0.060, 0.030)	0.517	0.027 (−0.029, 0.083)	0.350
	Model 3	−0.013 (−0.061, 0.034)	0.578	0.019 (−0.038, 0.075)	0.516
	Model 4	0.002 (−0.047, 0.052)	0.931	0.018 (−0.039, 0.074)	0.537
CIMT (*n* = 1730)	Model 1	−0.011 (−0.020, −0.002)	0.022 *	−0.013 (−0.025, 0.0001)	0.053
	Model 2	−0.008 (−0.018, 0.001)	0.080	−0.009 (−0.022, 0.004)	0.171
	Model 3	−0.007 (−0.017, 0.003)	0.153	−0.008 (−0.021, 0.005)	0.124
	Model 4	−0.004 (−0.015, 0.007)	0.466	−0.008 (−0.021, 0.005)	0.203
PWV (log) (*n* = 1707)	Model 1	0.009 (0.002, 0.016)	0.014 *	0.008 (−0.001, 0.017)	0.084
	Model 2	0.007 (0.001, 0.014)	0.045	0.006 (−0.004, 0.015)	0.219
	Model 3	0.004 (−0.004, 0.011)	0.319	0.003 (−0.006, 0.013)	0.527
	Model 4	0.001 (−0.007, 0.009)	0.849	0.003 (−0.006, 0.013)	0.484
AIx (log) (*n* = 1631)	Model 1	0.043 (0.020, 0.065)	<0.001 *	0.029 (−0.002, 0.060)	0.063
	Model 2	0.043 (0.020, 0.066)	<0.001 *	0.029 (−0.002, 0.060)	0.063
	Model 3	0.039 (0.014, 0.063)	0.002 *	0.024 (−0.007, 0.055)	0.125
	Model 4	0.032 (0.006, 0.057)	0.018 *	0.025 (−0.006, 0.056)	0.111

AIx, Augmentation Index; BMI, body mass index; CIMT, Carotid Intima-Media Thickness; CMD, cardiometabolic diseases; DBP, diastolic blood pressure; HDL-C, high-density lipoprotein cholesterol; HOMA-IR, homeostasis model assessment insulin resistance; LDL-C, low-density lipoprotein cholesterol; SD, standard deviation; SBP, systolic blood pressure; PWV, Pulse Wave Velocity; WC, waist circumference. Data were analysed for 3184 males for all CVD risk factors. For CIMT, PWV, and AIx, data were analysed for 1730, 1707, and 1631 males, respectively; 1 SD of total meat for males for all CVD risk factors was equal to 27.72 g/day; 1 SD total meat for males for CIMT was equal to 25.87 g/day; 1 SD total meat for males for PWV was equal to 25.95 g/day; 1 SD total meat for males for AIx was equal to 26.35 g/day; * significant association after accounting for the effect of multiple testing using the Benjamini–Hochberg method. ^a^ β-coefficient of 0.31 means that a 1 SD (27.72 g/day) increase in meat intake among male participants was associated with 0.31 kg/m^2^ higher BMI after adjusting for the effect of potential confounders on BMI. ^b^ β-coefficient of 0.43 means that a 10 g per 1000 kcal per day increase in meat intake among male participants was associated with 0.43 kg/m^2^ higher BMI after adjusting for the effect of potential confounders on BMI.

**Table 3:** Association between chicken and red meat intake with CMD risk factors and measures of subclinical atherosclerosis among males: β-coefficients (95% confidence interval) per SD change of red meat and chicken intake.

CVD Risk Factor	Regression Models	Red Meat		Chicken	
		β-Coef (95% CI)	*p*-Value	β-Coef (95% CI)	*p*-Value
SBP (*n* = 3183)	Model 1	0.52 (−0.04, 1.07)	0.068	0.33 (−0.24, 0.89)	0.255
	Model 2	0.35 (−0.21, 0.90)	0.221	0.22 (−0.34, 0.78)	0.443
	Model 3	0.25 (−0.32, 0.81)	0.394	0.15 (−0.42, 0.72)	0.605
	Model 4	0.19 (−0.38, 0.76)	0.514	0.06 (−0.53, 0.76)	0.856
DBP (*n* = 3183)	Model 1	0.81 (0.35, 1.26)	0.001	0.45 (−0.01, 0.91)	0.056
	Model 2	0.63 (0.17, 1.09)	0.007	0.34 (−0.13, 0.80)	0.153
	Model 3	0.53 (0.07, 0.99)	0.026	0.27 (−0.19, 0.74)	0.252
	Model 4	0.48 (0.01, 0.94)	0.046	0.18 (−0.30, 0.66)	0.452
BMI (*n* = 3183)	Model 1	0.29 (0.16, 0.43)	<0.001	0.37 (0.23, 0.50)	<0.001
	Model 2	0.20 (0.07, 0.33)	0.002	0.31 (0.17, 0.44)	<0.001
	Model 3	0.19 (0.06, 0.32)	0.004	0.31 (0.18, 0.44)	<0.001
	Model 4	0.14 ^a^ (0.01, 0.27)	0.035	0.22 ^b^ (0.09, 0.36)	0.001
WC (*n* = 3183)	Model 1	9.30 (5.70, 12.89)	<0.001	8.93 (5.28, 12.58)	<0.001
	Model 2	6.46 (2.95, 9.98)	<0.001	6.97 (3.41, 10.53)	<0.001
	Model 3	5.94 (2.39, 9.50)	0.001	6.90 (3.31, 10.49)	<0.001
	Model 4	4.66 (1.09, 8.23)	0.010	4.69 (1.03, 8.35)	0.012
Fasting Glucose (log) (*n* = 3183)	Model 1	0.005 (−0.001, 0.011)	0.096	−0.006 (−0.012, 0.001)	0.089
	Model 2	0.004 (−0.003, 0.010)	0.265	−0.007 (−0.013, −0.001)	0.038
	Model 3	0.004 (−0.003, 0.010)	0.226	−0.006 (−0.013, 0.000)	0.051
	Model 4	0.004 (−0.002, 0.011)	0.204	−0.006 (−0.012, 0.001)	0.073
HOMA-IR (log) (*n* = 3183)	Model 1	0.017 (−0.018, 0.051)	0.337	0.045 (0.010, 0.080)	0.013
	Model 2	0.003 (−0.032, 0.037)	0.873	0.036 (0.001, 0.071)	0.042
	Model 3	0.005 (−0.029, 0.040)	0.771	0.036 (0.001, 0.071)	0.047
	Model 4	0.002 (−0.033, 0.036)	0.932	0.029 (−0.007, 0.065)	0.110
Total cholesterol (*n* = 3183)	Model 1	0.87 (−0.48, 2.21)	0.206	2.88 (1.52, 4.25)	<0.001
	Model 2	0.51 (−0.83, 1.86)	0.455	2.68 (1.32, 4.05)	<0.001
	Model 3	0.45 (0.91, 1.82)	0.515	2.75 (1.36, 4.13)	<0.001
	Model 4	0.20 (−1.17, 1.58)	0.774	2.31 (0.90, 3.73)	0.001
HDL-C (*n* = 3183)	Model 1	−0.59 (−1.05, −0.13)	0.011	0.34 (−0.13, 0.81)	0.154
	Model 2	−0.47 (−0.93, −0.01)	0.045	0.44 (−0.03, 0.91)	0.068
	Model 3	−0.46 (−0.93, 0.003)	0.051	0.48 (0.01, 0.96)	0.046
	Model 4	−0.48 (−0.95, −0.01)	0.045	0.45 (−0.03, 0.94)	0.068
LDL-C (*n* = 3183)	Model 1	0.58 (−0.54, 1.71)	0.309	2.00 (0.85, 3.14)	0.001
	Model 2	0.20 (0.93, 1.32)	0.729	1.77 (0.63, 2.91)	0.002
	Model 3	0.19 (−0.95, 1.33)	0.747	1.82 (0.67, 2.98)	0.002
	Model 4	0.06 (−1.09, 1.21)	0.100	1.59 (0.41, 2.78)	0.008
TG (log) (*n* = 3183)	Model 1	0.033 (0.016, 0.051)	<0.001	0.024 (0.006, 0.042)	0.009
	Model 2	0.030 (0.012, 0.047)	0.001	0.021 (0.003, 0.039)	0.020
	Model 3	0.028 (0.010, 0.046)	0.003	0.021 (0.002, 0.039)	0.028
	Model 4	0.024 (0.006, 0.043)	0.009	0.015 (−0.004, 0.034)	0.116
CRP (log) (*n* = 3183)	Model 1	0.003 (−0.045, 0.051)	0.902	−0.004 (−0.052, 0.045)	0.890
	Model 2	−0.009 (−0.057, 0.040)	0.725	−0.009 (−0.058, 0.039)	0.706
	Model 3	−0.009 (−0.058, 0.040)	0.718	−0.008 (−0.057, 0.042)	0.758
	Model 4	−0.003 (−0.052, 0.047)	0.926	0.004 (−0.047, 0.055)	0.880
CIMT (*n* = 1730)	Model 1	−0.005 (−0.016, 0.005)	0.323	−0.007 (−0.018, 0.004)	0.189
	Model 2	−0.004 (−0.014, 0.007)	0.530	−0.006 (−0.017, 0.005)	0.262
	Model 3	−0.002 (−0.013, 0.009)	0.718	−0.006 (−0.017, 0.005)	0.272
	Model 4	−0.001 (−0.012, 0.010)	0.877	−0.004 (−0.015, 0.008)	0.547
PWV (log) (*n* = 1707)	Model 1	0.007 (−0.001, 0.015)	0.079	0.004 (−0.004, 0.012)	0.367
	Model 2	0.006 (−0.002, 0.014)	0.143	0.003 (−0.005, 0.011)	0.468
	Model 3	0.003 (−0.005, 0.011)	0.421	0.002 (−0.007, 0.010)	0.714
	Model 4	0.002 (−0.006, 0.010)	0.581	−0.001 (−0.009, 0.007)	0.835
AIx (log) (*n* = 1631)	Model 1	0.025 (−0.001, 0.050)	0.059	0.025 (−0.001, 0.052)	0.058
	Model 2	0.025 (−0.001, 0.051)	0.056	0.026 (0.000, 0.052)	0.052
	Model 3	0.023 (−0.003, 0.049)	0.086	0.023 (−0.003, 0.050)	0.088
	Model 4	0.021 (−0.006, 0.047)	0.128	0.017 (−0.010, 0.045)	0.214

AIx, Augmentation Index; BMI, body mass index; CIMT, Carotid Intima-Media Thickness; CMD, cardiometabolic diseases; DBP, diastolic blood pressure; HDL-C, high-density lipoprotein cholesterol; HOMA-IR, homeostasis model assessment insulin resistance; LDL-C, low-density lipoprotein cholesterol; SD, standard deviation; SBP, systolic blood pressure; PWV, Pulse Wave Velocity; WC, waist circumference. Data were analysed for 3184 males for all CMD risk factors. For CIMT, PWV, and AIx, data were analysed for 1730, 1707 and 1631 males, respectively. Results from secondary analyses were undertaken as exploratory analyses with *p*-value < 0.05 as significant; 1 SD of red meat for males for all CVD risk factors was equal to 9.74 g/day; 1 SD of red meat for males for CIMT was equal to 9.15 g/day; 1 SD of red meat for males for PWV was equal to 9.14 g/day; 1 SD of red meat for males for AIx was equal to 9.25 g/day; 1 SD of chicken for males for all CVD risk factors was equal to 22.13 g/day; 1 SD of chicken for males for CIMT was equal to 19.98 g/day; 1 SD of chicken for males for PWV was equal to 20.15 g/day; 1 SD of chicken for males for AIx was equal to 20.46 g/day; ^a^ β-coefficient of 0.14 means that a 1 SD (9.74 g/day) increase in red meat intake among male participants was associated with 0.14 kg/m^2^ higher BMI after adjusting for the effect of potential confounders on BMI. ^b^ β-coefficient of 0.22 means that a 1 SD (22.13 g/day) increase in chicken intake among male participants was associated with 0.22 kg/m^2^ higher BMI after adjusting for the effect of potential confounders on BMI.

**Table 4:** Association between total meat intake and CMD risk factors and measures of subclinical atherosclerosis among all females: β-coefficients (95% confidence interval) per SD change and 10 g per 1000 kcal per day of meat intake.

CVD Risk Factor	Regression Models	Per SD Change		10 g/1000 kcal/Day	
		β-Coef (95% CI)	*p*-Value	β-Coef (95% CI)	*p*-Value
SBP (*n* = 2828)	Model 1	0.52 (0.05, 0.99)	0.030	1.19 (0.49, 1.88)	<0.001 *
	Model 2	0.45 (−0.02, 0.93)	0.062	1.11 (0.41, 1.81)	0.002 *
	Model 3	0.41 (−0.10, 0.92)	0.116	1.08 (0.38, 1.79)	0.003 *
	Model 4	0.51 (−0.01, 1.02)	0.056	1.04 (0.33, 1.75)	0.004 *
DBP (*n* = 2828)	Model 1	0.51 (0.12, 0.90)	0.010 *	0.93 (0.35, 1.51)	0.002 *
	Model 2	0.43 (0.04, 0.83)	0.033	0.84 (0.26, 1.42)	0.005 *
	Model 3	0.41 (−0.01, 0.83)	0.057	0.82 (0.24, 1.41)	0.006 *
	Model 4	0.47 (0.04, 0.90)	0.032	0.80 (0.21, 1.39)	0.008 *
BMI (*n* = 2828)	Model 1	0.26 (0.12, 0.40)	<0.001 *	0.27 (0.06, 0.47)	0.013 *
	Model 2	0.17 (0.03, 0.31)	0.018 *	0.17 (−0.04, 0.37)	0.117
	Model 3	0.12 (−0.03, 0.27)	0.124	0.15 (−0.06, 0.35)	0.166
	Model 4	0.11 (−0.05, 0.26)	0.179	0.16 (−0.05, 0.37)	0.132
WC (*n* = 2828)	Model 1	7.17 (3.72, 10.62)	<0.001 *	6.60 (1.48, 11.72)	0.012 *
	Model 2	5.06 (1.62, 8.51)	0.004 *	4.19 (−0.89, 9.27)	0.106
	Model 3	3.29 (−0.38, 6.97)	0.079	3.43 (−1.65, 8.52)	0.186
	Model 4	2.66 (−1.08, 6.41)	0.163	3.98 (−1.12, 9.09)	0.126
Fasting Glucose (log) (*n* = 2828)	Model 1	0.004 (−0.002, 0.010)	0.193	0.013 (0.004, 0.022)	0.004 *
	Model 2	0.002 (−0.004, 0.008)	0.498	0.011 (0.002, 0.020)	0.016 *
	Model 3	0.005 (−0.002, 0.011)	0.143	0.012 (0.003, 0.021)	0.007 *
	Model 4	0.006 (−0.001, 0.013)	0.070	0.012 (0.003, 0.021)	0.011 *
HOMA-IR (log) (*n* = 2828)	Model 1	0.057 (0.026, 0.088)	<0.001 *	0.087 (0.041, 0.132)	<0.001 *
	Model 2	0.042 (0.011, 0.073)	0.008 *	0.071 (0.025, 0.116)	0.002 *
	Model 3	0.045 (0.012, 0.078)	0.007 *	0.072 (0.026, 0.118)	0.002 *
	Model 4	0.044 (0.010, 0.077)	0.011 *	0.075 (0.029, 0.121)	0.001 *
Total cholesterol (*n* = 2828)	Model 1	2.08 (0.82, 3.33)	0.001 *	2.98 (1.12, 4.84)	0.002 *
	Model 2	1.96 (0.69, 3.23)	0.002 *	2.84 (0.97, 4.71)	0.003 *
	Model 3	2.20 (0.84, 3.55)	0.002 *	2.89 (1.01, 4.77)	0.003 *
	Model 4	2.22 ^a^ (0.84, 3.61)	0.002 *	2.97 ^b^ (1.08, 4.86)	0.002 *
HDL-C (*n* = 2828)	Model 1	0.10 (−0.35, 0.56)	0.655	−0.35 (−1.02, 0.33)	0.306
	Model 2	0.19 (−0.27, 0.64)	0.426	−0.26 (−0.94, 0.41)	0.444
	Model 3	0.14 (−0.35, 0.63)	0.579	−0.32 (−1.00, 0.36)	0.352
	Model 4	0.06 (−0.44, 0.56)	0.804	−0.27 (−0.95, 0.41)	0.439
LDL-C (*n* = 2828)	Model 1	1.64 (0.59, 2.68)	0.002 *	2.48 (0.93, 4.03)	0.002 *
	Model 2	1.46 (0.41, 2.52)	0.007 *	2.28 (0.72, 3.83)	0.004 *
	Model 3	1.61 (0.48, 2.73)	0.005 *	2.33 (0.77, 3.89)	0.003 *
	Model 4	1.67 (0.52, 2.82)	0.004 *	2.36 (0.79, 3.93)	0.003 *
TG (log) (*n* = 2828)	Model 1	0.015 (−0.001, 0.031)	0.063	0.032 (0.008, 0.056)	0.008 *
	Model 2	0.015 (−0.001, 0.031)	0.072	0.032 (0.008, 0.056)	0.009 *
	Model 3	0.019 (0.002, 0.036)	0.032	0.033 (0.010, 0.057)	0.006 *
	Model 4	0.019 (0.002, 0.037)	0.032	0.034 (0.010, 0.058)	0.006 *
CRP (log) (*n* = 2828)	Model 1	0.053 (0.007, 0.099)	0.024 *	0.047 (−0.021, 0.115)	0.173
	Model 2	0.036 (−0.011, 0.082)	0.130	0.028 (−0.041, 0.096)	0.427
	Model 3	0.043 (−0.006, 0.093)	0.088	0.031 (−0.038, 0.099)	0.381
	Model 4	0.037 (−0.013, 0.088)	0.146	0.036 (−0.033, 0.104)	0.306
CIMT (*n* = 1573)	Model 1	0.001 (−0.008, 0.010)	0.778	0.004 (−0.010, 0.017)	0.586
	Model 2	0.003 (−0.007, 0.012)	0.607	0.005 (−0.008, 0.019)	0.483
	Model 3	0.004 (−0.007, 0.014)	0.493	0.006 (−0.008, 0.019)	0.436
	Model 4	0.004 (−0.007, 0.014)	0.500	0.006 (−0.008, 0.020)	0.424
PWV (log) (*n* = 1570)	Model 1	−0.006 (−0.013, 0.001)	0.079	−0.010 (−0.020, 0.0001)	0.047
	Model 2	−0.007 (−0.014, −0.0001)	0.048	−0.011 (−0.021, −0.001)	0.034
	Model 3	−0.006 (−0.013, 0.002)	0.137	−0.009 (−0.019, 0.001)	0.075
	Model 4	−0.005 (−0.013, 0.003)	0.189	−0.010 (−0.020, −0.000)	0.050
AIx (log) (*n* = 1480)	Model 1	0.022 (−0.0006, 0.0450)	0.057	0.016 (−0.016, 0.049)	0.316
	Model 2	0.023 (−0.0005, 0.0455)	0.055	0.017 (−0.016, 0.049)	0.314
	Model 3	0.018 (−0.007, 0.043)	0.150	0.012 (−0.020, 0.045)	0.458
	Model 4	0.018 (−0.007, 0.043)	0.154	0.013 (−0.020, 0.046)	0.440

AIx, Augmentation Index; BMI, body mass index; CIMT, Carotid Intima-Media Thickness; CMD, cardiometabolic diseases; DBP, diastolic blood pressure; HDL-C, high-density lipoprotein cholesterol; HOMA-IR, homeostasis model assessment insulin resistance; LDL-C, low-density lipoprotein cholesterol; SD, standard deviation; SBP, systolic blood pressure; PWV, Pulse Wave Velocity; WC, waist circumference. Data were analysed for 2828 females for all CVD risk factors. For CIMT, PWV, and AIx, data were analysed for 1573, 1570, and 1480 females, respectively. To mitigate the risk of type 1 errors due to performing multiple statistical tests, we reported *p* values which were significant at a false discovery rate of 5% using the Benjamini–Hochberg method. For this, we considered all models with the same exposure as part of the same family of tests (i.e., 14 tests per family); * significant association after accounting for the effect of multiple testing using the Benjamini–Hochberg method; 1 SD of total meat for females for all CVD risk factors was equal to 14.28 g/day; 1 SD of total meat for females for CIMT was equal to 14.9 g/day; 1 SD of total meat for females for PWV was equal to 14.9 g/day; 1 SD of total meat for females for AIx was equal to 15.04 g/day; ^a^ β-coefficient of 2.22 means that a 1 SD (14.28 g/day) increase in meat intake among female participants was associated with 2.22 mg/dL higher total cholesterol after adjusting for the effect of potential confounders on total cholesterol. ^b^ β-coefficient of 2.97 means that a 10 g per 1000 kcal per day increase in meat intake among female participants was associated with 2.97 mg/dL higher total cholesterol after adjusting for the effect of potential confounders on total cholesterol.

**Table 5:** Association between chicken and red meat intake with CMD risk factors and measures of subclinical atherosclerosis among females: β-coefficients (95% confidence interval) per SD change of red meat and chicken intake.

CVD Risk Factor	Regression Models	Red Meat		Chicken	
		β-Coef (95% CI)	*p*-Value	β-Coef (95% CI)	*p*-Value
SBP (*n* = 2828)	Model 1	0.13 (−0.35, 0.61)	0.599	0.48 (−0.01, 0.97)	0.054
	Model 2	0.08 (−0.40, 0.56)	0.735	0.44 (−0.05, 0.93)	0.079
	Model 3	0.05 (−0.44, 0.54)	0.856	0.42 (−0.09, 0.92)	0.107
	Model 4	0.09 (−0.41, 0.58)	0.729	0.49 (−0.02, 1.00)	0.060
DBP (*n* = 2828)	Model 1	0.26 (−0.14, 0.65)	0.207	0.37 (−0.04, 0.78)	0.074
	Model 2	0.21 (−0.20, 0.61)	0.316	0.32 (−0.09, 0.73)	0.124
	Model 3	0.19 (−0.22, 0.59)	0.374	0.31 (−0.11, 0.73)	0.144
	Model 4	0.21 (−0.20, 0.62)	0.311	0.36 (−0.07, 0.78)	0.096
BMI (*n* = 2828)	Model 1	0.15 (0.01, 0.29)	0.040	0.17 (0.03, 0.32)	0.022
	Model 2	0.09 (−0.05, 0.24)	0.192	0.12 (−0.03, 0.26)	0.114
	Model 3	0.07 (−0.08, 0.21)	0.360	0.08 (−0.07, 0.23)	0.294
	Model 4	0.06 (−0.08, 0.21)	0.404	0.07 (−0.08, 0.22)	0.366
WC (*n* = 2828)	Model 1	4.06 (0.57, 7.55)	0.023	4.81 (1.24, 8.38)	0.008
	Model 2	2.71 (−0.76, 6.19)	0.126	3.52 (−0.03, 7.06)	0.052
	Model 3	1.72 (−1.82, 5.25)	0.342	2.33 (−1.31, 5.97)	0.209
	Model 4	1.44 (−2.11, 4.99)	0.426	1.85 (−1.84, 5.52)	0.326
Fasting Glucose (log) (*n* = 2828)	Model 1	0.002 (−0.004, 0.008)	0.451	0.003 (−0.004, 0.009)	0.409
	Model 2	0.001 (−0.005, 0.007)	0.360	0.002 (−0.005, 0.008)	0.460
	Model 3	0.003 (−0.004, 0.009)	0.405	0.003 (−0.003, 0.010)	0.309
	Model 4	0.003 (−0.003, 0.009)	0.317	0.004 (−0.002, 0.011)	0.193
HOMA-IR (log) (*n* = 2828)	Model 1	−0.002 (−0.034, 0.029)	0.883	0.065 (0.033, 0.100)	<0.001
	Model 2	−0.012 (−0.044, 0.020)	0.443	0.056 (0.024, 0.088)	0.001
	Model 3	−0.012 (−0.044, 0.020)	0.477	0.058 (0.026, 0.091)	<0.001
	Model 4	−0.012 (−0.044, 0.020)	0.449	0.057 (0.024, 0.090)	0.001
Total cholesterol (*n* = 2828)	Model 1	0.36 (−0.90, 1.63)	0.572	2.02 (0.73, 3.31)	0.002
	Model 2	0.29 (−0.98, 1.55)	0.660	1.95 (0.66, 3.25)	0.003
	Model 3	0.37 (−0.93, 1.66)	0.581	2.13 (0.80, 3.50)	0.002
	Model 4	0.38 ^a^ (−0.92, 1.68)	0.570	2.15 ^b^ (0.80, 3.51)	0.002
HDL-C (*n* = 2828)	Model 1	0.00 (−0.45, 0.46)	0.993	0.11 (−0.35, 0.58)	0.635
	Model 2	0.05 (−0.40, 0.51)	0.819	0.16 (−0.30, 0.63)	0.491
	Model 3	0.03 (−0.43, 0.50)	0.892	0.13 (−0.35, 0.61)	0.604
	Model 4	0.00 (−0.47, 0.47)	1.000	0.07 (−0.42, 0.56)	0.781
LDL-C (*n* = 2828)	Model 1	0.29 (−0.76, 1.34)	0.587	1.59 (0.51, 2.67)	0.004
	Model 2	0.18 (−0.88, 1.23)	0.330	1.48 (0.40, 2.56)	0.007
	Model 3	0.21 (−0.87, 1.28)	0.707	1.61 (0.50, 2.72)	0.005
	Model 4	0.24 (−0.85, 1.31)	0.671	1.66 (0.54, 2.78)	0.004
TG (log) (*n* = 2828)	Model 1	0.000 (−0.017, 0.016)	0.980	0.017 (0.000, 0.033)	0.046
	Model 2	−0.001 (−0.017, 0.016)	0.957	0.017 (0.000, 0.032)	0.049
	Model 3	0.002 (−0.015, 0.019)	0.839	0.020 (0.002, 0.037)	0.027
	Model 4	0.002 (−0.015, 0.019)	0.822	0.020 (0.002, 0.037)	0.026
CRP (log) (*n* = 2828)	Model 1	0.041 (−0.006, 0.088)	0.085	0.027 (−0.021, 0.075)	0.267
	Model 2	0.029 (−0.018, 0.076)	0.219	0.017 (−0.030, 0.064)	0.487
	Model 3	0.034 (−0.014, 0.082)	0.163	0.022 (−0.027, 0.071)	0.383
	Model 4	0.032 (−0.017, 0.080)	0.197	0.017 (−0.032, 0.067)	0.493
CIMT (*n* = 1573)	Model 1	−0.0001 (−0.011, 0.009)	0.810	0.002 (−0.008, 0.013)	0.643
	Model 2	0.000 (−0.010, 0.010)	0.958	0.003 (−0.007, 0.013)	0.574
	Model 3	0.001 (−0.009, 0.011)	0.868	0.003 (−0.007, 0.014)	0.545
	Model 4	0.001 (−0.009, 0.011)	0.869	0.003 (−0.007, 0.014)	0.550
PWV (log) (*n* = 1570)	Model 1	−0.002 (−0.009, 0.006)	0.637	−0.006 (−0.013, 0.002)	0.146
	Model 2	−0.002 (−0.010, 0.005)	0.524	−0.006 (−0.014, 0.002)	0.116
	Model 3	−0.002 (−0.009, 0.006)	0.679	−0.005 (−0.013, 0.003)	0.197
	Model 4	−0.001 (−0.009, 0.006)	0.724	−0.005 (−0.012, 0.003)	0.250
AIx (log) (*n* = 1480)	Model 1	−0.007 (−0.030, 0.017)	0.587	0.029 (0.005, 0.053)	0.019
	Model 2	−0.007 (−0.030, 0.017)	0.593	0.029 (0.005, 0.053)	0.018
	Model 3	−0.010 (−0.034, 0.015)	0.441	0.026 (0.001, 0.051)	0.039
	Model 4	−0.010 (−0.034, 0.015)	0.443	0.026 (0.001, 0.052)	0.040

AIx, Augmentation Index; BMI, body mass index; CIMT, Carotid Intima-Media Thickness; CMD, cardiometabolic diseases; DBP, diastolic blood pressure; HDL-C, high-density lipoprotein cholesterol; HOMA-IR, homeostasis model assessment insulin resistance; LDL-C, low-density lipoprotein cholesterol; SD, standard deviation; SBP, systolic blood pressure; PWV, Pulse Wave Velocity; WC, waist circumference. Data were analysed for 2828 females for all CVD risk factors. For CIMT, PWV, and AIx, data were analysed for 1573, 1570 and 1480 females, respectively. Results from secondary analyses were undertaken as exploratory analyses, with *p*-value < 0.05 as significant; 1 SD of red meat for females for all CVD risk factors was equal to 6.42 g/day; 1 SD of red meat for females for CIMT was equal to 6.03 g/day; 1 SD of red meat for females for PWV was equal to 6.01 g/day; 1 SD of red meat for females for AIx was equal to 6.02 g/day; 1 SD of chicken for females for all CVD risk factors was equal to 10.86 g/day; 1 SD of chicken for females for CIMT was equal to 11.61 g/day; 1 SD of chicken for females for PWV was equal to 11.65 g/day; 1 SD of chicken for females for AIx was equal to 11.78 g/day; ^a^ β-coefficient of 0.38 means that a 1 SD (6.42 g/day) increase in red meat intake among female participants was associated with 0.38 mg/dL higher total cholesterol after adjusting for the effect of potential confounders on total cholesterol. ^b^ β-coefficient of 2.15 means that a 1 SD (10.86 g/day) increase in chicken intake among female participants was associated with 2.15 mg/dL higher total cholesterol after adjusting for the effect of potential confounders on total cholesterol.

## Data Availability

The raw data supporting the conclusions of this article will be made available by the authors on request.

## Supplementary Materials

The following supporting information can be downloaded at: https://www.mdpi.com/article/10.3390/nu16050746/s1, Table S1: Association between total meat intake and CVD risk factors and measures of subclinical atherosclerosis among males who were consuming meat: β-coefficients (95% confidence interval) per SD change and 10 g per 1000 kcal per day of meat intake; Table S2: Association between total meat intake and CVD risk factors and measures of subclinical atherosclerosis among females who were consuming meat: β-coefficients (95% confidence interval) per SD change and 10 g per 1000 kcal per day of meat intake. Table S3: Association between total meat intake and CVD risk factors and measures of subclinical atherosclerosis among males with no diagnosis of CVD or DM or hypertension: β-coefficients (95% confidence interval) per SD change and 10 g per 1000 kcal per day of meat intake; Table S4: Association between total meat intake and CVD risk factors and measures of subclinical atherosclerosis among females with no diagnosis of CVD or DM or hypertension: β-coefficients (95% confidence interval) per SD change and 10 g per 1000 kcal per day of meat intake.
