# Supplementary material for: The Association of Total Meat Intake with Cardio-Metabolic Disease Risk Factors and Measures of Sub-Clinical Atherosclerosis in an Urbanising Community of Southern India: A Cross-Sectional Analysis for the APCAPS Cohort
Source: Nutrients. 2024 Mar 5;16(5):746. doi: 10.3390/nu16050746 (PMC10934090; doi:10.3390/nu16050746)
Supplement: Supplementary file 1 [file nutrients-16-00746-s001.zip › nutrients-2888250-supplementary.pdf]

Supplementary Table S1: Association between total meat intake and CVD risk factors and measures of subclinical atherosclerosis among *males* who were consuming meat: β-coefficients (95% confidence interval) per SD change and 10 gram per 1000 kcal per day of meat intake

CVD risk factor	Regression Models	Per SD change		10 gram/1000 kcal/day	
		β-coef (95% CI)	p-value	β-coef (95% CI)	p-value
SBP, (n= 3097)	Model 1	0.68 (0.16, 1.21)	0.011*	1.01 (0.35, 1.67)	0.003*
	Model 2	0.45 (−0.08, 0.98)	0.099	0.73 (0.07, 1.39)	0.032
	Model 3	0.31 (−0.25, 0.87)	0.276	0.54 (−0.13, 1.20)	0.114
	Model 4	0.18 (−0.41, 0.76)	0.550	0.56 (-0.11, 1.22)	0.101
DBP, (n= 3097)	Model 1	1.01 (0.58, 1.44)	<0.001*	1.20 (0.66, 1.74)	<0.001*
	Model 2	0.75 (0.32, 1.19)	0.001*	0.89 (0.35, 1.44)	0.001*
	Model 3	0.62 (0.16, 1.08)	0.008*	0.73 (0.18, 1.28)	0.010*
	Model 4	0.49 (0.01, 0.97)	0.048	0.75 (0.20, 1.30)	0.007*
BMI, (n= 3097)	Model 1	0.56 (0.44, 0.69)	<0.001*	0.64 (0.48, 0.80)	<0.001*
	Model 2	0.44 (0.32, 0.56)	<0.001*	0.49 (0.33, 0.64)	<0.001*
	Model 3	0.44 (0.31, 0.57)	<0.001*	0.44 (0.28, 0.59)	<0.001*
	Model 4	0.32[a] (0.19, 0.46)	<0.001*	0.46[b] (0.31, 0.61)	<0.001*
WC, (n= 3097)	Model 1	15.22 (11.84, 18.61)	<0.001*	17.12 (12.86, 21.38)	<0.001*
	Model 2	11.26 (7.91, 14.60)	<0.001*	12.25 (8.06, 16.45)	<0.001*
	Model 3	10.85 (7.33, 14.37)	<0.001*	10.74 (6.54, 14.94)	<0.001*
	Model 4	7.88 (4.21, 11.55)	<0.001*	11.27 (7.10, 15.44)	<0.001*
Fasting Glucose (log), (n= 3097)	Model 1	−0.001 (−0.006, 0.005)	0.871	−0.002 (−0.005, 0.009)	0.667
	Model 2	−0.003 (−0.009, 0.003)	0.188	−0.002 (−0.009, 0.006)	0.713
	Model 3	−0.003 (−0.009, 0.004)	0.415	−0.002 (−0.010, 0.005)	0.536
	Model 4	−0.003 (−0.009, 0.004)	0.454	−0.002 (−0.010, 0.005)	0.528
HOMA-IR (log), (n= 3097)	Model 1	0.062 (0.029, 0.094)	0.001*	0.079 (0.038, 0.120)	0.001*
	Model 2	0.044 (0.011, 0.077)	0.010*	0.057 (0.015, 0.098)	0.007*
	Model 3	0.046 (0.011, 0.080)	0.009	0.048 (0.007, 0.089)	0.022*
	Model 4	0.037 (0.001, 0.073)	0.045	0.050 (0.009, 0.091)	0.017*
Total cholesterol, (n= 3097)	Model 1	3.23 (1.97, 4.89)	<0.001*	4.12 (2.54, 5.71)	<0.001*
	Model 2	2.76 (1.48, 4.03)	<0.001*	3.55 (1.95, 5.15)	<0.001*
	Model 3	2.89 (1.55, 4.24)	<0.001*	3.23 (1.62, 4.84)	<0.001*
	Model 4	2.32 (0.91, 3.72)	0.001*	3.35 (1.74, 4.95)	<0.001*
HDL	Model 1	−0.18 (−0.62, 0.26)	0.418	0.005 (−0.54, 0.56)	0.985
	Model 2	0.003 (−0.44, 0.45)	0.990	0.23 (−0.33, 0.78)	0.424
	Model 3	0.08 (−0.38, 0.55)	0.729	0.32 (−0.23, 0.88)	0.255
	Model 4	0.07 (−0.42, 0.56)	0.781	0.33 (−0.23, 0.89)	0.251
LDL	Model 1	2.22 (1.17, 3.28)	<0.001*	3.07 (1.75, 4.40)	<0.001*
	Model 2	1.71 (0.65, 2.78)	0.002*	2.47 (1.13, 3.81)	<0.001*
	Model 3	1.82 (0.70, 2.94)	0.002*	2.20 (0.86, 3.55)	0.001*
	Model 4	1.52 (0.34, 2.70)	0.011*	2.26 (0.92, 3.61)	0.001*
TG (log)	Model 1	0.047 (0.030, 0.064)	<0.001*	0.046 (0.025, 0.066)	<0.001*
	Model 2	0.041 (0.024, 0.058)	<0.001*	0.038 (0.017, 0.059)	<0.001*
	Model 3	0.040 (0.022, 0.058)	<0.001*	0.033 (0.012, 0.054)	0.003*
	Model 4	0.032 (0.012, 0.050)	0.001*	0.034 (0.013, 0.056)	0.002*
CRP (log)	Model 1	−0.001 (−0.046, 0.044)	0.966	0.045 (−0.012, 0.102)	0.121
	Model 2	−0.015 (−0.061, 0.031)	0.525	0.029 (−0.028, 0.087)	0.316
	Model 3	−0.011 (−0.059, 0.038)	0.662	0.022 (−0.035, 0.080)	0.449
	Model 4	0.005 (−0.046, 0.055)	0.859	0.020 (−0.037, 0.078)	0.492
CIMT (n= 1682)	Model 1	−0.010 (−0.019, -0.001)	0.040	−0.011 (−0.023, 0.002)	0.099
	Model 2	−0.007 (−0.016, 0.002)	0.145	−0.007 (−0.020, 0.006)	0.306
	Model 3	−0.005 (−0.015, 0.005)	0.297	−0.005 (−0.018, 0.008)	0.409
	Model 4	−0.002 (−0.013, 0.009)	0.690	−0.006 (−0.019, 0.007)	0.360
PWV (log)	Model 1	0.009 (0.002, 0.016)	0.009*	0.010 (−0.0001, 0.019)	0.052
	Model 2	0.008 (0.001, 0.015)	0.029*	0.007 (−0.003, 0.017)	0.148
	Model 3	0.005 (-0.003, 0.012)	0.233	0.004 (−0.005, 0.014)	0.382
	Model 4	0.002 (-0.006, 0.010)	0.675	0.005 (−0.005, 0.015)	0.323
AIx (log)	Model 1	0.042 (0.019, 0.065)	<0.001*	0.027 (−0.004, 0.058)	0.084
	Model 2	0.043 (0.020, 0.067)	<0.001*	0.028 (−0.003, 0.060)	0.079
	Model 3	0.036 (0.011, 0.061)	0.004*	0.023 (−0.009, 0.054)	0.159

	Model 4	0.029 (0.003, 0.056)	0.028*	0.025 (–0.007, 0.056)	0.131

SD, standard deviation; SBP, systolic blood pressure; DBP, diastolic blood pressure; CIMT, Carotid Intima-Media Thickness; PWV, Pulse Wave Velocity; AIx, Augmentation Index; BMI, body mass index; WC, waist circumference; HDL-C, high density lipoprotein cholesterol; LDL-C, low density lipoprotein cholesterol; HOMA-IR, Homeostasis model assessment insulin resistance;
Data was analysed for 3097 males for all CVD risk factors. For CIMT, PWV, and AIx, data was analyzed for 1682, 1658 and 1584 males, respectively;
1 SD of total meat for male for all CVD risk factors was equal to 27.79 grams/day; 1 SD total meat for male for CIMT was equal to 25.92 grams/day; 1 SD total meat for male for PWV was equal to 26.00 grams/day; 1 SD total meat for male for AIx was equal to 26.67 grams/day; * significant association after accounting for the effect of multiple testing using the Benjamini–Hochberg method; [a]β-coefficient of 0.32 means that a 1 SD (27.79 grams/day) increase in meat intake among male participants was associated with 0.32 kg/m^2 higher BMI after adjusting for the effect of potential confounders on BMI. [b]β-coefficient of 0.46 means that a-10 gram per 1000 kcal per day increase in meat intake among male participants was associated with 0.46 kg/m^2 higher BMI after adjusting for the effect of potential confounders on BMI.

Supplementary Table S2: Association between total meat intake and CVD risk factors and measures of subclinical atherosclerosis among *females* who were consuming meat: β-coefficients (95% confidence interval) per SD change and 10 gram per 1000 kcal per day of meat intake

CVD risk factor	Regression Models	Per SD change		10 gram/1000 kcal/day	
		β-coef (95% CI)	p-value	β-coef (95% CI)	p-value
SBP, (n= 2675)	Model 1	0.48 (–0.01, 0.97)	0.053	1.15 (0.42, 1.87)	0.002*
	Model 2	0.41 (–0.08, 0.91)	0.100	1.07 (0.34, 1.80)	0.004*
	Model 3	0.35 (–0.19, 0.88)	0.201	1.03 (0.29, 1.76)	0.006*
	Model 4	0.43 (–0.112, 0.97)	0.120	0.98 (0.24, 1.72)	0.009*
DBP, (n= 2675)	Model 1	0.46 (0.05, 0.86)	0.027	0.85 (0.25, 1.45)	0.006*
	Model 2	0.38 (–0.03, 0.78)	0.072	0.76 (0.15, 1.36)	0.014*
	Model 3	0.34 (–0.10, 0.79)	0.128	0.73 (0.12, 1.34)	0.019*
	Model 4	0.39 (–0.06, 0.84)	0.090	0.71 (0.10, 1.33)	0.023*
BMI, (n= 2675)	Model 1	0.28 (0.13, 0.42)	<0.001*	0.29 (0.07, 0.50)	0.009*
	Model 2	0.18 (0.04, 0.33)	0.015	0.18 (–0.04, 0.39)	0.106
	Model 3	0.13 (–0.02, 0.29)	0.094	0.15 (–0.06, 0.37)	0.165
	Model 4	0.12 (–0.04, 0.28)	0.152	0.17 (–0.04, 0.39)	0.116
WC, (n= 2675)	Model 1	7.04 (3.47, 10.61)	<0.001*	6.14 (0.81, 11.47)	0.024*
	Model 2	4.72 (1.15, 8.29)	0.010*	3.42 (–1.88, 8.72)	0.205
	Model 3	3.04 (–0.81, 6.89)	0.122	2.56 (–2.75, 7.87)	0.345
	Model 4	2.33 (–1.59, 6.25)	0.243	3.30 (–2.04, 8.65)	0.226
Fasting Glucose (log), (n= 2675)	Model 1	0.004 (–0.003, 0.010)	0.276	0.012 (0.003, 0.022)	0.008*
	Model 2	0.002 (–0.005, 0.008)	0.647	0.010 (0.001, 0.019)	0.031
	Model 3	0.004 (–0.003, 0.011)	0.247	0.011 (0.002, 0.021)	0.018*
	Model 4	0.005 (–0.002, 0.012)	0.136	0.011 (0.001, 0.020)	0.028
HOMA-IR (log), (n= 2675)	Model 1	0.056 (0.024, 0.087)	0.001*	0.085 (0.038, 0.132)	<0.001*
	Model 2	0.040 (0.008, 0.071)	0.013*	0.067 (0.020, 0.114)	0.005*
	Model 3	0.045 (0.010, 0.079)	0.011*	0.068 (0.021, 0.115)	0.005*
	Model 4	0.041 (0.007, 0.076)	0.019	0.073 (0.025, 0.120)	0.003*
Total cholesterol, (n= 2675)	Model 1	2.21 (0.91, 3.51)	0.001*	3.21 (1.27, 5.14)	0.001*
	Model 2	2.06 (0.75, 3.37)	0.002*	3.02 (1.07, 4.97)	0.002*
	Model 3	2.21 (0.79, 3.63)	0.002*	2.98 (1.02, 4.94)	0.003*
	Model 4	2.21[a] (0.77, 3.66)	0.003*	3.10[b] (1.12, 5.07)	0.002*
HDL, (n= 2675)	Model 1	0.16 (–0.31, 0.62)	0.507	–0.29 (–0.98, 0.41)	0.419
	Model 2	0.24 (–0.23, 0.71)	0.326	–0.21 (–0.90, 0.49)	0.567
	Model 3	0.18 (–0.33, 0.69)	0.492	–0.26 (–0.96, 0.44)	0.474
	Model 4	0.11 (–0.41, 0.62)	0.684	–0.19 (–0.90, 0.51)	0.594
LDL, (n= 2675)	Model 1	1.75 (0.67, 2.83)	0.002*	2.68 (1.07, 4.29)	0.001*
	Model 2	1.55 (0.46, 2.64)	0.005*	2.44 (0.82, 4.06)	0.003*
	Model 3	1.64 (0.46, 2.82)	0.006*	2.42 (0.79, 4.04)	0.004*
	Model 4	1.68 (0.48, 2.88)	0.006*	2.47 (0.83, 4.11)	0.003*
TG (log), (n= 2675)	Model 1	0.015 (–0.002, 0.031)	0.081	0.032 (0.007, 0.056)	0.011*
	Model 2	0.015 (–0.002, 0.031)	0.089	0.032 (0.007, 0.056)	0.012*
	Model 3	0.018 (0.000, 0.036)	0.053	0.032 (0.007, 0.057)	0.011*
	Model 4	0.018 (–0.001, 0.036)	0.058	0.034 (0.008, 0.059)	0.009*
CRP (log), (n= 2675)	Model 1	0.054 (0.007, 0.101)	0.024	0.049 (–0.021, 0.120)	0.169
	Model 2	0.036 (–0.012, 0.083)	0.143	0.028 (–0.043, 0.098)	0.443
	Model 3	0.047 (–0.004, 0.099)	0.071	0.031 (–0.040, 0.101)	0.393
	Model 4	0.041 (–0.012, 0.093)	0.127	0.039 (–0.033, 0.110)	0.291
CIMT (n= 1493)	Model 1	–0.0003 (–0.010, 0.010)	0.961	0.001 (–0.013, 0.016)	0.842

	Model 2	0.001 (–0.009, 0.011)	0.812	0.003 (–0.011, 0.017)	0.688
	Model 3	0.002 (–0.009, 0.013)	0.699	0.003 (–0.011, 0.018)	0.642
	Model 4	0.002 (–0.009, 0.013)	0.699	0.004 (–0.011, 0.018)	0.637
PWV (log) (n= 1493)	Model 1	–0.006 (–0.013, 0.002)	0.139	–0.009 (–0.019, 0.001)	0.089
	Model 2	–0.006 (–0.014, 0.001)	0.090	–0.010 (–0.020, 0.001)	0.066
	Model 3	–0.005 (–0.013, 0.003)	0.225	–0.008 (–0.019, 0.002)	0.119
	Model 4	–0.004 (–0.012, 0.004)	0.298	–0.009 (–0.020, 0.001)	0.082
AI (log) (n= 1411)	Model 1	0.024 (0.0003, 0.0469)	0.047	0.018 (–0.015, 0.051)	0.277
	Model 2	0.025 (0.001, 0.048)	0.040	0.019 (–0.014, 0.052)	0.262
	Model 3	0.023 (–0.002, 0.048)	0.076	0.016 (–0.017, 0.049)	0.342
	Model 4	0.024 (–0.002, 0.050)	0.071	0.016 (–0.017, 0.050)	0.340

SD, standard deviation; SBP, systolic blood pressure; DBP, diastolic blood pressure; CIMT, Carotid Intima-Media Thickness; PWV, Pulse Wave Velocity; AIx, Augmentation Index; BMI, body mass index; WC, waist circumference; HDL-C, high density lipoprotein cholesterol; LDL-C, low density lipoprotein cholesterol; HOMA-IR, Homeostasis model assessment insulin resistance;

Data was analysed for 2675 females for all CVD risk factors. For CIMT, PWV, and AIx, data was analyzed for 1493, 1493, and 1411 females, respectively; 1 SD of total meat for female for all CVD risk factors was equal to 14.72 grams/day; 1 SD total meat for female for CIMT was equal to 14.93 grams/day; 1 SD total meat for female for PWV was equal to 14.93 grams/day; 1 SD total meat for female for AIx was equal to 15.07 grams/day; * significant association after accounting for the effect of multiple testing using the Benjamini–Hochberg method;

[a]β-coefficient of **2.21** means that a 1 SD (14.28 grams/day) increase in meat intake among female participants was associated with 2.21 mg/dL higher total cholesterol after adjusting for the effect of potential confounders on total cholesterol.

[b]β-coefficient of **3.10** means that a-10 gram per 1000 kcal per day increase in meat intake among female participants was associated with 3.10 mg/dL higher total cholesterol after adjusting for the effect of potential confounders on total cholesterol.

Supplementary Table S3. Association between total meat intake and CVD risk factors and measures of subclinical atherosclerosis among *males* with no diagnosis of CVD or DM or hypertension: β-coefficients (95% confidence interval) per SD change and 10 gram per 1000 kcal per day of meat intake

CVD risk factor	Regression Models	Per SD change		10 gram/1000 kcal/day	
		β-coef (95% CI)	p-value	β-coef (95% CI)	p-value
SBP, (n= 2861)	Model 1	0.79 (0.26, 1.31)	0.003*	1.18 (0.52, 1.84)	<0.001*
	Model 2	0.58 (0.06, 1.10)	0.028	0.95 (0.28, 1.61)	0.005
	Model 3	0.44 (–0.11, 0.99)	0.114	0.74 (0.39, 1.41)	0.029
	Model 4	0.28 (–0.29, 0.86)	0.335	0.75 (0.09, 1.41)	0.027
DBP, (n= 2779)	Model 1	1.07 (0.63, 1.51)	<0.001*	1.32 (0.77, 1.87)	<0.001*
	Model 2	0.80 (0.37, 1.24)	<0.001*	1.04 (0.49, 1.59)	<0.001*
	Model 3	0.68 (0.23, 1.14)	0.003*	0.86 (0.31, 1.42)	0.002*
	Model 4	0.53 (0.06, 1.01)	0.028	0.87 (0.32, 1.43)	0.002*
BMI, (n= 2861)	Model 1	0.51 (0.38, 0.63)	<0.001*	0.59 (0.43, 0.74)	<0.001*
	Model 2	0.39 (0.27, 0.52)	<0.001*	0.45 (0.29, 0.61)	<0.001*
	Model 3	0.40 (0.27, 0.53)	<0.001*	0.41 (0.25, 0.57)	<0.001*
	Model 4	0.28 (0.15, 0.42)	<0.001*	0.42 (0.26, 0.57)	<0.001*
WC, (n= 3184)	Model 1	13.74 (10.38, 17.11)	<0.001*	15.40 (11.09, 19.71)	<0.001*
	Model 2	10.15 (6.82, 13.47)	<0.001*	11.16 (6.92, 15.40)	<0.001*
	Model 3	10.01 (6.53, 13.49)	<0.001*	9.85 (5.61, 14.09)	<0.001*
	Model 4	6.89 (3.26, 10.51)	<0.001*	10.05 (5.85, 14.26)	<0.001*
Fasting Glucose (log), (n= 2861)	Model 1	–0.005 (–0.006, 0.005)	0.840	–0.001 (–0.007, 0.006)	0.970
	Model 2	–0.002 (–0.007, 0.003)	0.478	–0.002 (–0.009, 0.005)	0.540
	Model 3	–0.002 (–0.007, 0.004)	0.519	–0.002 (–0.009, 0.004)	0.464
	Model 4	–0.002 (–0.008, 0.004)	0.459	–0.002 (–0.009, 0.004)	0.466
HOMA-IR (log), (n= 2861)	Model 1	0.048 (0.017, 0.080)	0.003*	0.062 (0.021, 0.102)	0.003*
	Model 2	0.033 (0.001, 0.064)	0.044*	0.044 (0.003, 0.084)	0.036
	Model 3	0.037 (0.004, 0.070)	0.029	0.040 (–0.001, 0.080)	0.053
	Model 4	0.027 (–0.007, 0.062)	0.124	0.041 (0.001, 0.080)	0.049
Total cholesterol, (n= 2861)	Model 1	3.34 (2.08, 4.61)	<0.001*	4.57 (2.95, 6.19)	<0.001*
	Model 2	2.88 (1.60, 4.16)	<0.001*	4.03 (2.39, 5.66)	<0.001*
	Model 3	3.04 (1.70, 4.39)	<0.001*	3.71 (2.07, 5.35)	<0.001*
	Model 4	2.45 (1.04, 3.85)	0.001*	3.76 (2.12, 5.39)	<0.001*
HDL, (n= 2861)	Model 1	0.009 (–0.42, 0.44)	0.969	0.29 (–0.26, 0.84)	0.299
	Model 2	0.16 (–0.28, 0.59)	0.477	0.46 (–0.91, 1.02)	0.101
	Model 3	0.23 (–0.23, 0.68)	0.329	0.52 (–0.03, 1.08)	0.065
	Model 4	0.19 (–0.29, 0.66)	0.441	0.53 (–0.03, 1.08)	0.063
LDL, (n= 2861)	Model 1	2.34 (1.28, 3.40)	<0.001*	3.45 (2.10, 4.80)	<0.001*

	Model 2	1.87 (0.80, 2.94)	0.001*	2.92 (1.56, 4.28)	<0.001*
	Model 3	2.02 (0.90, 3.14)	<0.001*	2.67 (1.31, 4.04)	<0.001*
	Model 4	1.71 (0.54, 2.89)	0.004*	2.69 (1.33, 4.06)	0.001*
TG (log), (n= 2861)	Model 1	0.041 (0.025, 0.058)	<0.001*	0.040 (0.019, 0.061)	<0.001*
	Model 2	0.036 (0.019, 0.053)	<0.001*	0.033 (0.012, 0.055)	<0.001*
	Model 3	0.034 (0.017, 0.052)	<0.001*	0.028 (0.007, 0.050)	0.010*
	Model 4	0.027 (0.008, 0.045)	0.005*	0.029 (0.007, 0.051)	0.009*
CRP (log), (n= 2861)	Model 1	0.002 (–0.044, 0.049)	0.918	0.049 (–0.010, 0.108)	0.106
	Model 2	–0.010 (–0.057, 0.036)	0.664	0.035 (–0.025, 0.095)	0.252
	Model 3	–0.007 (–0.056, 0.042)	0.772	0.028 (–0.032, 0.097)	0.367
	Model 4	0.007 (–0.045, 0.059)	0.791	0.027 (–0.033, 0.087)	0.379
CIMT, (n= 1539)	Model 1	–0.011 (–0.020, –0.002)	0.027*	–0.015 (–0.029, -0.002)	0.022
	Model 2	–0.007 (–0.016, 0.002)	0.133	–0.011 (–0.024, 0.002)	0.107
	Model 3	–0.007 (–0.017, 0.003)	0.192	–0.010 (–0.023, 0.003)	0.147
	Model 4	–0.004 (–0.015, 0.007)	0.440	–0.010 (–0.023, 0.003)	0.141
PWV (log), (n= 1517)	Model 1	0.010 (0.003, 0.017)	0.007*	0.010 (–0.001, 0.020)	0.055
	Model 2	0.008 (0.001, 0.016)	0.023	0.008 (–0.002, 0.018)	0.133
	Model 3	0.005 (–0.003, 0.012)	0.244	0.004 (–0.006, 0.014)	0.404
	Model 4	0.001 (–0.007, 0.009)	0.828	0.005 (–0.006, 0.015)	0.378
AI (log), (n= 1447)	Model 1	0.047 (0.023, 0.070)	<0.001*	0.036 (0.003, 0.069)	0.033
	Model 2	0.048 (0.024, 0.071)	<0.001*	0.036 (0.003, 0.070)	0.034
	Model 3	0.042 (0.016, 0.068)	0.001*	0.029 (–0.004, 0.063)	0.088
	Model 4	0.033 (0.006, 0.061)	0.016*	0.030 (–0.004, 0.063)	0.083

AIx, Augmentation Index; BMI, body mass index; CIMT, Carotid Intima-Media Thickness; CMD, cardiometabolic diseases; DBP, diastolic blood pressure; HDL-C, high density lipoprotein cholesterol; HOMA-IR, Homeostasis model assessment insulin resistance; LDL-C, low density lipoprotein cholesterol; SD, standard deviation; SBP, systolic blood pressure; PWV, Pulse Wave Velocity; WC, waist circumference; Data was analysed for 3184 males for all CVD risk factors. For CIMT, PWV, and AIx, data was analyzed for 1730, 1707, and 1631 males, respectively; 1 SD of total meat for male for all CVD risk factors was equal to 27.72 grams/day; 1 SD total meat for male for CIMT was equal to 25.87 grams/day; 1 SD total meat for male for PWV was equal to 25.95 grams/day; 1 SD total meat for male for AIx was equal to 26.35 grams/day; *significant association after accounting for the effect of multiple testing using the Benjamini-Hochberg method.

Supplementary Table S4. Association between total meat intake and CVD risk factors and measures of subclinical atherosclerosis among *females* with no diagnosis of CVD or DM or hypertension: β-coefficients (95% confidence interval) per SD change and 10 gram per 1000 kcal per day of meat intake

CVD risk factor	Regression Models	Per SD change		10 gram/1000 kcal/day	
		β-coef (95% CI)	p-value	β-coef (95% CI)	p-value
SBP, (n= 2496)	Model 1	0.55 (0.07, 1.03)	0.024	1.20 (0.49, 1.92)	<0.001*
	Model 2	0.48 (–0.01, 0.96)	0.051	1.13 (0.41, 1.85)	0.002*
	Model 3	0.42 (–0.10, 0.93)	0.111	1.09 (0.37, 1.81)	0.003*
	Model 4	0.51 (–0.02, 1.03)	0.057	1.05 (0.32, 1.77)	<0.001*
DBP, (n= 2496)	Model 1	0.51 (0.10, 0.91)	0.014*	0.91 (0.30, 1.51)	0.003*
	Model 2	0.43 (0.02, 0.83)	0.039	0.83 (0.22, 1.43)	0.007*
	Model 3	0.42 (–0.02, 0.85)	0.060	0.82 (0.21, 1.42)	0.009*
	Model 4	0.47 (0.03, 0.91)	0.037	0.80 (0.18, 1.40)	0.011*
BMI, (n= 2496)	Model 1	0.23 (0.09, 0.38)	0.002*	0.23 (0.02, 0.45)	0.039*
	Model 2	0.16 (0.01, 0.30)	0.033*	0.15 (–0.07, 0.36)	0.183
	Model 3	0.11 (–0.05, 0.26)	0.168	0.13 (–0.09, 0.34)	0.251
	Model 4	0.10 (–0.06, 0.26)	0.224	0.14 (–0.08, 0.36)	0.211
WC, (n= 2496)	Model 1	6.77 (3.24, 10.29)	<0.001*	5.86 (0.55, 11.17)	0.030*
	Model 2	4.96 (1.45, 8.48)	0.006*	3.88 (–1.39, 9.15)	0.149
	Model 3	3.23 (–0.52, 6.98)	0.091	3.08 (–2.20, 8.35)	0.253
	Model 4	2.60 (–1.22, 6.42)	0.182	3.65 (–1.65, 8.94)	0.177
Fasting Glucose (log), (n= 2496)	Model 1	0.005 (–0.001, 0.011)	0.094	0.013 (0.004, 0.021)	0.004*
	Model 2	0.003 (–0.002, 0.009)	0.255	0.011 (0.002, 0.020)	0.012*
	Model 3	0.005 (–0.001, 0.011)	0.106	0.012 (0.003, 0.020)	0.008*
	Model 4	0.006 (–0.001, 0.013)	0.076	0.012 (0.003, 0.020)	0.009*

HOMA-IR (log), (n= 2496)	Model 1	0.049 (0.019, 0.079)	0.001*	0.079 (0.033, 0.123)	0.001*
	Model 2	0.037 (0.007, 0.067)	0.017*	0.065 (0.019, 0.110)	0.005*
	Model 3	0.037 (0.005, 0.069)	0.023*	0.064 (0.018, 0.109)	0.006*
	Model 4	0.036 (0.003, 0.068)	0.032*	0.066 (0.021, 0.112)	0.004*
Total cholesterol, (n= 2496)	Model 1	2.36 (1.07, 3.64)	<0.001*	3.20 (1.26, 5.13)	0.001*
	Model 2	2.20 (0.91, 3.50)	0.001*	3.01 (1.07, 4.95)	0.002*
	Model 3	2.45 (1.07, 3.83)	0.001*	3.02 (1.08, 4.97)	0.002*
	Model 4	2.45 (1.04, 3.86)	0.001*	3.13 (1.17, 5.08)	0.002*
HDL, (n= 2496)	Model 1	0.23 (–0.22, 0.68)	0.309	–0.07 (–0.75, 0.61)	0.832
	Model 2	0.30 (–0.16, 0.75)	0.198	–0.01 (–0.69, 0.67)	0.979
	Model 3	0.34 (–0.15, 0.83)	0.169	–0.02 (–0.70, 0.67)	0.959
	Model 4	0.25 (–0.24, 0.75)	0.314	0.05 (–0.63, 0.74)	0.879
LDL, (n= 2496)	Model 1	1.85 (0.78, 2.91)	0.001*	2.62 (1.01, 4.23)	0.001*
	Model 2	1.65 (0.57, 2.72)	0.003*	2.39 (0.78, 4.00)	0.004*
	Model 3	1.78 (0.64, 2.93)	0.002*	2.40 (0.78, 4.01)	0.004*
	Model 4	1.83 (0.66, 3.00)	0.002*	2.44 (0.82, 4.06)	0.003*
TG (log), (n= 2496)	Model 1	0.014 (–0.002, 0.030)	0.091	0.027 (0.002, 0.052)	0.032*
	Model 2	0.014 (–0.002, 0.030)	0.098	0.027 (0.002, 0.051)	0.034*
	Model 3	0.015 (–0.003, 0.032)	0.096	0.026 (0.002, 0.051)	0.037*
	Model 4	0.016 (–0.002, 0.034)	0.082	0.026 (0.002, 0.051)	0.038*
CRP (log), (n= 2496)	Model 1	0.055 (0.007, 0.103)	0.025*	0.047 (–0.025, 0.119)	0.202
	Model 2	0.042 (–0.007, 0.090)	0.091	0.032 (–0.040, 0.105)	0.382
	Model 3	0.048 (–0.004, 0.099)	0.070	0.034 (–0.039, 0.107)	0.358
	Model 4	0.041 (–0.011, 0.094)	0.124	0.040 (–0.033, 0.113)	0.280
CIMT, (n= 1375)	Model 1	0.003 (–0.006, 0.013)	0.498	0.003 (–0.012, 0.017)	0.715
	Model 2	0.005 (–0.005, 0.015)	0.353	0.004 (–0.011, 0.018)	0.605
	Model 3	0.006 (–0.005, 0.016)	0.307	0.004 (–0.010, 0.019)	0.586
	Model 4	0.005 (–0.006, 0.015)	0.404	0.005 (–0.009, 0.020)	0.479
PWV (log), (n= 1365)	Model 1	–0.007 (–0.014, 0.001)	0.053	–0.013 (–0.023, –0.002)	0.021
	Model 2	–0.008 (–0.015, –0.001)	0.034	–0.013 (–0.024, –0.003)	0.016
	Model 3	–0.007 (–0.015, 0.001)	0.079	–0.012 (–0.023, –0.001)	0.030
	Model 4	–0.007 (–0.015, 0.002)	0.109	–0.013 (-0.023, –0.002)	0.018
AI (log), (n= 1285)	Model 1	0.021 (–0.003, 0.045)	0.087	0.017 (–0.018, 0.051)	0.335
	Model 2	0.022 (–0.003, 0.046)	0.083	0.017 (–0.018, 0.052)	0.332
	Model 3	0.020 (–0.006, 0.046)	0.133	0.015 (–0.020, 0.050)	0.402
	Model 4	0.020 (–0.006, 0.047)	0.130	0.015 (–0.020, 0.051)	0.393

AIx, Augmentation Index; BMI, body mass index; CIMT, Carotid Intima-Media Thickness; CMD, cardiometabolic diseases; DBP, diastolic blood pressure; HDL-C, high density lipoprotein cholesterol; HOMA-IR, Homeostasis model assessment insulin resistance; LDL-C, low density lipoprotein cholesterol; SD, standard deviation; SBP, systolic blood pressure; PWV, Pulse Wave Velocity; WC, waist circumference; Data was analysed for 2828 females for all CVD risk factors. For CIMT, PWV, and AIx, data was analyzed for 1573, 1570, and 1480 females, respectively; *significant association after accounting for the effect of multiple testing using the Benjamini-Hochberg method; 1 SD of total meat for female for all CVD risk factors was equal to 14.28 grams/day;1-sd total meat for female for CIMT was equal to 14.9 grams/day; 1 SD total meat for female for PWV was equal to 14.9 grams/day; 1 SD total meat for female for AIx was equal to 15.04 grams/day;
